# Physics-paired stimulated Raman scattering microscopy enables label-free phenotyping of lipid droplets 3D motility in live cells

**DOI:** 10.1038/s41377-026-02435-x

**Published:** 2026-07-24

**Authors:** Shulang Lin, Bin He, Chang Liu, Rongxuan Li, Le Xin, Zhiwei Huang

**Affiliations:** 1 Optical Bioimaging Laboratory, Department of Biomedical Engineering, College of Design and Engineering, National University of Singapore, Singapore, Singapore; 2 National University of Singapore (Suzhou) Research Institute, Suzhou, China; 3 National University of Singapore Guangzhou Research Translation and Innovation Institute, Guangzhou, Guangdong, China; 4 NUS Graduate School for Integrative Sciences and Engineering Programme (ISEP), National University of Singapore, Singapore, Singapore

**Keywords:** Imaging and sensing, Biophotonics

## Abstract

High-speed volumetric stimulated Raman scattering (SRS) microscopy offers unique capabilities for label-free chemical imaging in living systems, yet its performance is fundamentally constrained by the trade-off between imaging speed and signal-to-noise ratio (SNR). At the short pixel dwell times required for three-dimensional dynamic imaging, photon-limited detection leads to severe noise that cannot be effectively mitigated by existing denoising approaches, owing to the lack of ground truth data and temporally redundant measurements in live-cell conditions. Here we present PHYSIQ, a physics-paired in-phase and quadrature SRS imaging framework that fundamentally redefines data acquisition for noise-limited optical microscopy. By exploiting the intrinsic quadrature nature of heterodyne detection, PHYSIQ-SRS simultaneously acquires two spatially co-registered and temporally near-synchronous SRS image channels with statistically independent shot noise. This physics-paired measurement enables fully self-supervised Noise2Noise restoration without requiring ground truth or temporal redundancy. The implementation integrates dual-channel lock-in detection with defocus-corrected spatial co-registration and controlled temporal offset, establishing a robust and generalizable strategy for generating unbiased training pairs directly from physical measurements. This innovative approach achieves an SNR enhancement of ~12.5 dB while preserving quantitative Raman contrast, effectively overcoming the conventional speed-sensitivity limitation in volumetric SRS microscopy. The improved performance enables video-rate volumetric imaging and label-free 3D tracking of lipid droplets (LDs) in living cells. Using this capability, we uncover that LD dynamics are governed by discrete motility states with condition-dependent transitions, including spatial redistribution under nutrient perturbation, selective suppression of long-range transport upon glycolytic inhibition, and phase-dependent reprogramming during mitosis. PHYSIQ-SRS establishes a new paradigm of physics-enabled self-supervised imaging, providing a general solution to shot-noise-limited detection in laser-scanning microscopy. This advance opens new opportunities for high-speed, label-free volumetric imaging and quantitative investigation of live-cell biology, metabolic phenotyping, developmental imaging, and biomedical discovery.

## Abstract (web summary)

PHYSIQ-SRS transforms shot-noise-limited microscopy by pairing quadrature signals for fully self-supervised denoising, boosting SNR by 12.5 dB and enabling video-rate, label-free 3D imaging of dynamic lipid metabolism in living cells.

## Graphical abstract

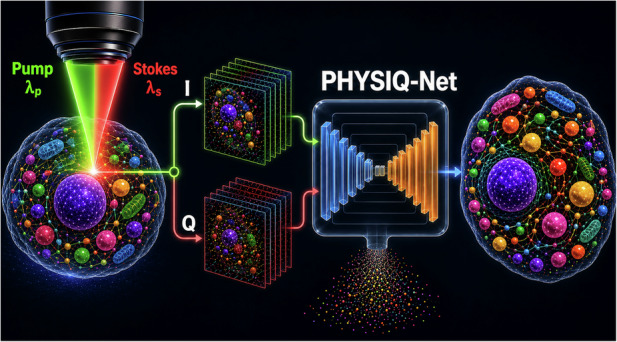

## Introduction

Lipid droplets (LDs) are not passive lipid stores but dynamic organelles that coordinate energy balance, membrane homeostasis and stress adaptation through continual interactions with other intracellular compartments^1,2^. Because these functions depend on where LDs are positioned and how they move, resolving LD 3D trafficking in living cells offers a direct window into metabolic organization in space and time^3–5^. Yet, despite the biological importance of LD dynamics, achieving high-speed, non-invasive 3D tracking of LDs remains technically challenging. Fluorescence microscopy can visualize LDs with high sensitivity^6^, but exogenous labels may perturb lipid biology. Extended recordings are further limited by bleaching, phototoxicity and the finite photon budget of fluorophores, especially when fast volumetric imaging is required. Holotomography provides a label-free route for 3D live-cell imaging, but its contrast is primarily refractive-index based and does not inherently provide vibrational chemical specificity for lipid-rich structures^7–9^.

Stimulated Raman scattering (SRS) microscopy has emerged as a powerful label-free method for imaging LDs and other biomolecules of cells and tissue with chemical specificity^10–15^. For instance, by tuning the pump and Stokes lasers to target the CH₂ vibrational bond (2845 cm⁻¹), SRS can selectively visualize neutral lipid content in live cells without requiring exogenous dyes^16–20^, but fast volumetric SRS imaging is strongly limited by the signal-to-noise ratio (SNR) ^21,22^. Although the use of a spatial light modulator (SLM) or deformable mirror can enhance z-scanning speed electronically^23,24^, the overall volumetric imaging rate is still significantly constrained by the 2D frame rate, largely because of inadequate SNR. Consequently, achieving fast 3D SRS microscopy typically sacrifices in image quality with poor SNR or demands high excitation powers to improve SNR which may induce photodamage and phototoxicity owing to heating or reactive oxygen species and thus perturbs normal cell physiology^25^.

Computational denoising has emerged as an appealing route to relax this trade-off, but existing methods remain poorly matched to the acquisition statistics of high-speed SRS. Classical non-local denoisers such as BM3D exploit redundancy among similar patches^26^, yet their performance deteriorates when the signal approaches the noise floor and structural self-similarity becomes difficult to recover reliably. Deep-learning-based methods can in principle significantly improve the SNR, but most supervised approaches require registered low-noise targets that are difficult or impossible to obtain for fast live-cell volumetric imaging^27,28^. Self-supervised methods avoid this requirement, but they impose their own assumptions. Noise2Void and related blind-spot approaches rely on local independence of pixel noise^29^, an assumption that is violated in raster-scanned SRS because short pixel dwell times become comparable to the response time of lock-in detection, producing spatially correlated noise^30^. Noise2Noise and related paired-training frameworks instead require two independent noisy observations of the same underlying scene^31,32^. In practice, these pairs are often generated by splitting frames or alternating image sequences^33,34^, but this fundamental requirement for temporal consistency limits their utility in denoising high-speed recordings of dynamic biological processes, where such stable conditions between frames are often unmet^35^. Thus, while denoising has transformed many fluorescence-imaging workflows^36^, the low-SNR, high-speed, motion-sensitive regime of volumetric SRS exposes a gap between the assumptions of existing algorithms and the physics of the measurement.

In this work, we introduce PHYSIQ, a physics-paired in-phase and quadrature SRS framework that enables self-supervised denoising for SNR-limited volumetric live-cell imaging. By generating two near-simultaneous SRS measurements of the same focal volume with identical Raman signals but independent shot noise, PHYSIQ enables unbiased Noise2Noise restoration without ground-truth data, improving effective SNR by ~12.5 dB while preserving quantitative biochemical contrast. This capability allows label-free, rapid 3D tracking and motility-state analysis of LDs, revealing dynamic reorganization of LD trafficking during nutrient perturbation and mitosis. PHYSIQ-SRS thus provides a powerful framework for volumetric, state-resolved phenotyping of organelle dynamics and cellular metabolism in living systems.

## Results

### Working principle of PHYSIQ

**Fig. 1:**
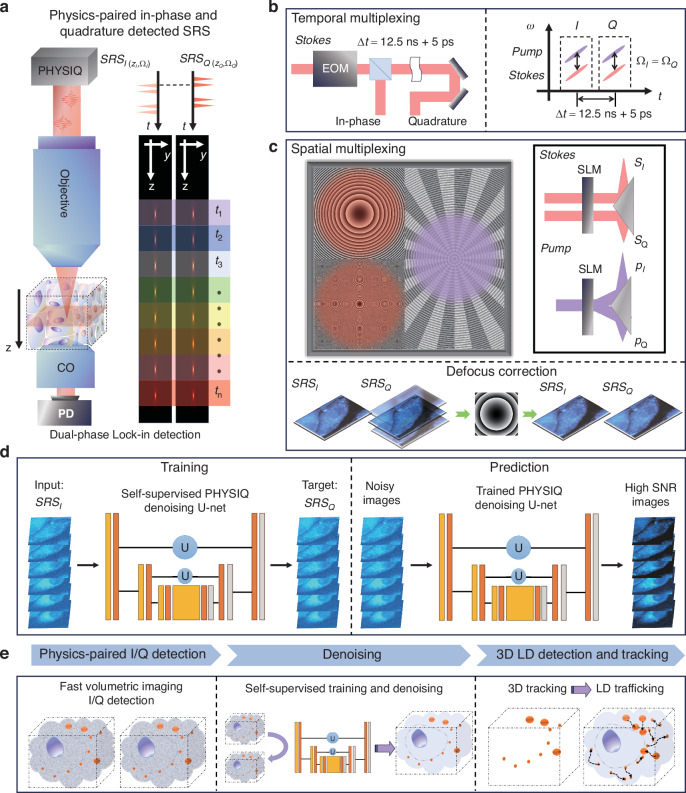
PHYSIQ strategy for physics-paired spatiotemporal multiplexing and self-supervised SRS denoising. **a** Schematic of the PHYSIQ-SRS imaging system. A physics-paired spatiotemporal multiplexing approach is used to simultaneously acquire two SRS images, *SRS*_*I*_ (*z*_*I*_*, Ω*_*I*_) and *SRS*_*Q*_ (*z*_*Q*_*, Ω*_*Q*_), corresponding to the in-phase (I) and quadrature (Q) channels. Here, ${z}_{I}$ and ${z}_{Q}$ denote the axial focal positions, and ${\Omega }_{I}$ and ${\Omega }_{Q}$ denote the corresponding Raman shifts. The two channels are spatially defocus-corrected to achieve matched focal planes while preserving statistically independent shot noise. **b** Temporal multiplexing scheme using an EOM to encode in-phase (*I*) and quadrature (*Q*) signals for simultaneous detection. **c** Spatial multiplexing with a single SLM that phase-modulates the pump and Stokes beams to generate two defocus-calibrated SRS channels with matched signal but independent shot noise realizations. **d** Self-supervised PHYSIQ denoising workflow. Paired low-SNR volumetric images from the I and Q channels are used as input–target pairs to train a 3D U-Net, enabling reconstruction of high-SNR SRS volumes without requiring clean ground-truth data. **e** Overall PHYSIQ workflow integrating simultaneous I/Q SRS acquisition, self-supervised denoising, and LD tracking for rapid, label-free 3D LD motility phenotyping

Figure 1a illustrates the operating principle of the PHYSIQ framework. Related quadrature SRS approaches have been employed to multiplex spectral and polarization information, including dual-phase SRS for simultaneous two-color imaging^19,37^ and polarization-resolved SRS for concurrent measurement of polarized and depolarized Raman tensor components^18^. Unlike these methods, PHYSIQ is designed to generate two simultaneously acquired SRS images from the same focal volume that contain an identical underlying Raman signal while maintaining statistically independent shot noise. This physics-paired acquisition strategy enables fully self-supervised denoising without requiring additional measurements, thereby making high-fidelity, high-speed volumetric SRS imaging feasible. Specifically, a mode-locked femtosecond laser (80 MHz repetition rate) provides the pump and Stokes pulses for SRS. The Stokes beam is intensity-modulated at ${f}_{M}=\,20{MHz}$ (${T}_{{EOM}}=50{ns}$) by an electro-optic modulator (EOM) and then split into two paths (denoted as the in-phase (I) and quadrature (Q) components) by a polarizing beam splitter (Fig. 1b). An optical delay of ($\Delta t=\frac{{T}_{{EOM}}}{4}=12.5\,{ns}$ delay) is introduced in one path so that the two channels are I/Q-orthogonal with respect to the lock-in reference and, because $\Delta t$ also equals one pulse period at 80 MHz, the two paths integrate disjoint subsets of laser pulses. A short sub-pulse offset $\delta t\approx 5{ps}\ll {T}_{{EOM}}$ is added to suppress residual intra-pulse interference without affecting I/Q demodulation^15,37^. To effectively resolve different chemical bonds, both the pump and Stokes pulses are linearly chirped using SF57 glass rods. This spectral-focusing scheme aligns the instantaneous frequencies of the two pulses, ensuring identical spectral-focusing bandwidths for the I and Q channels.

To create twin, diffraction-limited foci with preserved NA, a reflective phase-type SLM positioned conjugate to the objective back pupil is partitioned into three zones (Fig. 1c): one for the pump and two for the Stokes I/Q paths (see Supplementary Fig. S1). Appropriate phase masks steer and co-locate the pump focus with each Stokes focus in the sample. Residual axial mismatch is removed by applying a defocus phase in the pupil; a near-axis form is used ${\varphi }_{{defocus}}\left(r\right)\approx -\frac{k\Delta z}{2n{f}^{2}}{r}^{2}$, with $k=2\pi /\lambda$, *n* is refractive index, *f* is objective focal length, and *r* is pupil radius coordinate. This varifocal control enables per-channel defocus calibration and rapid z-scanning without mechanical motion. The pump and the two Stokes paths are then recombined and scanned across the sample to form twin, co-registered foci. The temporal multiplexing will produce two separate SRS signals that can be distinguished by phase-sensitive lock-in detection. After the condenser, SRS signals are filtered and directed to the photodiode. For a fixed Raman shift $\varOmega$ and depth $z$, the detected SRS signal *S(t)* at the photodiode can be written as

1 $$S\left(t\right)={{SRS}}_{I}\left(z\right)\sin \left(2\pi {f}_{M}t+{\varphi }_{0}\right)+{{SRS}}_{Q}\left(z\right)\cos \left(2\pi {f}_{M}t+{\varphi }_{0}\right)+n(t)$$

where SRS signals scale as ${{SRS}}_{\{I,Q\}}\propto \sigma (\Omega ){I}_{S}{I}_{p}$, with $\sigma$ denoting Raman cross-section, and ${I}_{P}$ and ${I}_{s}$ ${\rm{representing}}$ excitation light intensity of pump and Stokes beams. The term *n(t)* is the overall noise, heavily dominated by Poisson photon shot noise. Dual-phase lock-in detection with orthogonal local oscillators demodulates this signal into two image channels:

2 $${Y}_{I}={{SRS}}_{I}+{\varepsilon }_{I},{Y}_{Q}={{SRS}}_{Q}+{\varepsilon }_{Q}$$

where the residual noise terms, ${\varepsilon }_{I}$ and ${\varepsilon }_{Q}$, following low-pass filtering, are given by:

3 $${\varepsilon }_{I}=\frac{1}{T}{\int }_{0}^{T}n\left(t\right)\sin \left(2\pi {f}_{M}t\right){dt},{\varepsilon }_{Q}=\frac{1}{T}{\int }_{0}^{T}n\left(t\right)\cos \left(2\pi {f}_{M}t\right){dt}$$

Because the I and Q channels integrate mutually exclusive subsets of laser pulses, separated by (${T}_{{EOM}}/4$), and are demodulated at orthogonal phases, their noise components are statistically independent after bias normalization. Consequently, the residual noise has a zero mean and approximately zero cross-covariance between channels (see Supplementary Notes S1 and S3, Fig. S2):

4 $$E\left({\varepsilon }_{I}\right)=E\left({\varepsilon }_{Q}\right)=0,{Cov}({\varepsilon }_{I},{\varepsilon }_{Q})\approx 0$$

Residual common-mode fluctuations are strongly attenuated by the high-frequency MHz modulation and the narrow bandwidth of the lock-in filter. These physics-paired SRS images thus provide independent noisy realizations of the same underlying signal^38^, satisfying the fundamental assumptions required for full Noise2Noise training^31^.

This acquisition strategy differs fundamentally from a conventional beam-splitter approach, where the same photon stream is divided between two detectors, leading to partially correlated noise. In PHYSIQ, each channel receives a distinct, non-overlapping subset of photons, resulting in statistically independent noise realizations while preserving the same underlying Raman signal (Supplementary Notes S1–S3). Under shot-noise-limited conditions, the two channels are therefore expected to exhibit negligible cross-covariance. Although residual laser relative intensity noise (RIN) and electronic noise may introduce weak common-mode correlations^39^, we experimentally verified channel independence by acquiring paired images of static samples and quantifying pixel-wise noise correlations between channels. The measured correlations remained close to zero, even at the shortest dwell time of 0.5 μs per pixel, confirming that the paired images can be treated as effectively decorrelated observations suitable for Noise2Noise self-supervised denoising (Supplementary Note S3, Fig. S2).

We then exploited these physics-paired volumes to train a 3D U-Net in a fully self-supervised framework (Fig. 1d). One channel served as the network input and its paired counterpart as the training target, with channel assignments swapped during training to fully utilize the dataset. Although both channels are individually noisy, this paired formulation enables the network to learn the shared underlying signal while suppressing stochastic fluctuations, converging toward the latent structure of the sample.

As outlined in Fig. 1e, PHYSIQ subsequently transforms the restored volumetric SRS time series into quantitative biological readouts. Following denoising, enhanced contrast enables robust 3D segmentation of LDs and reliable temporal linking of individual droplets across frames. The resulting trajectories are used to extract kinematic descriptors, enabling downstream analysis of organelle dynamics. In this way, PHYSIQ connects physics-derived paired image formation with label-free organelle phenotyping, revealing motility-state-dependent remodeling of LD transport in living cells. We therefore term the framework PHYSIQ, as it establishes a physically grounded route to self-supervised denoising in volumetric SRS imaging without requiring clean ground truth, temporal averaging, or artificially separated acquisition pairs.

### PHYSIQ denoising performance and quantitative evaluation

**Fig. 2:**
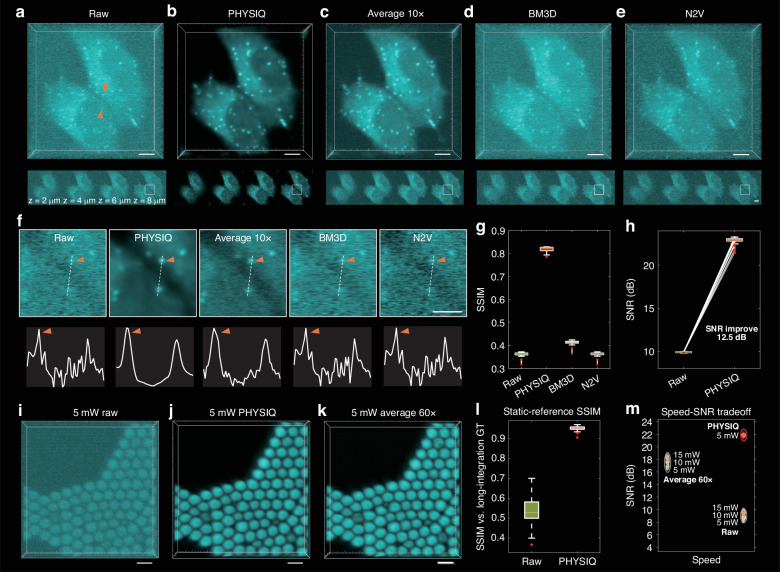
Denoising performance and quantitative evaluation of PHYSIQ. **a**–**e** Qualitative comparison of denoising performance on a representative 3D SRS volume of LDs in a fixed HeLa cell: raw input (**a**), PHYSIQ reconstruction (**b**), 10-frame temporal averaging (**c**), BM3D (**d**), and Noise2Void (N2V) (**e**). Top rows show maximum-intensity projections, and bottom rows show representative z-slice montages. **f** Enlarged LD regions (top) and corresponding intensity line profiles (bottom), demonstrating that PHYSIQ preserves LD morphology and sharp boundaries comparable to 10-frame temporal averaging. Box plots summarize measurements across multiple fields of view for LD regions of interest. **g** Structural similarity index (SSIM) and (**h**) signal-to-noise ratio (SNR) comparisons across methods, showing that PHYSIQ achieves ~12.5 dB SNR improvement over the raw input and substantially higher SSIM than all competing methods. **i**–**k** Static phantom validation using 3 μm polystyrene–poly(methyl methacrylate) (PS/PMMA) microspheres acquired at the Raman peak of 2900 cm⁻¹: low-SNR raw SRS image under low-power conditions (**i**), PHYSIQ reconstruction from the same low-power paired acquisition (**j**), and a high-SNR reference image acquired from the same field of view using the same excitation power (5 mW) with 60-frame temporal averaging (**k**). **l** SSIM calculated within bead-centered 3D regions of interest relative to the high-SNR reference. **m** Speed–SNR trade-off comparing single-volume acquisition, repeated frame averaging, and PHYSIQ reconstruction; each point represents an individual bead. Scale bar, 5 μm. Imaging conditions: 320 × 320 × 8 voxels; pixel dwell time, 4 μs; paired I/Q 2D frame time, 775 ms; 3D volume time, 6.2 s. For 10-frame averaging, the effective temporal integration window was 62 s

To evaluate PHYSIQ performance, we benchmarked it against the raw input, ten-frame temporal averaging, BM3D, and Noise2Void (N2V) using identical datasets acquired from a fixed HeLa cell (Fig. 2a-e). A single raw SRS volume exhibited low signal-to-noise ratio (SNR), rendering many LDs indistinguishable from the background (Fig. 2a). Temporal averaging of ten consecutive volumes reduced noise and revealed additional LDs, but at the cost of temporal integration, which may obscure dynamic structural information (Fig. 2c). In contrast, PHYSIQ reconstructed LDs from a single low-SNR volume into compact, high-contrast features with a substantially cleaner background, approaching the perceptual quality of the 10× averaged reference while preserving sharper droplet boundaries (Fig. 2b). Under identical conditions, BM3D provided only modest improvement (Fig. 2d), whereas N2V showed minimal measurable enhancement (Fig. 2e; Supplementary Note S4). Importantly, z-slice montages across the imaging depth (*z* = 2–8 μm; bottom row) demonstrate that PHYSIQ maintains high SNR and structural fidelity throughout the volumetric stack.

We next assessed local reconstruction fidelity at representative LDs planes (e.g., *z* = 8 μm; Fig. 2f). In the raw image, individual LDs were partially obscured by noise, with poorly resolved boundaries. PHYSIQ recovered these structures with peak intensities and peak-to-valley contrast closely matching the 10× averaged reference, while preserving sharper boundaries than temporal averaging. This distinction reflects the underlying denoising mechanisms: temporal averaging suppresses noise through signal integration over time, which can blur high-contrast edges despite preserving gross morphology, whereas PHYSIQ removes noise without temporal fusion and thus retains dynamic structural detail. By comparison, BM3D and N2V failed to recover LDs above the noise floor. BM3D relies on non-local patch redundancy, which becomes ineffective under extremely low-SNR conditions, while N2V assumes local noise independence—an assumption violated in fast SRS imaging, where lock-in detector time constants approach the raster pixel dwell time. These results demonstrate that PHYSIQ substantially improves image quality without compromising either spatial or temporal resolution.

To enable reference-based quantitative evaluation without requiring long-exposure ground truth, we leveraged the minimal positional drift of LDs over tens of seconds (Supplementary Video 1) and used the average of the first 10 volumes as an auxiliary reference for consistency analysis. Structural similarity index (SSIM) measurements between each single-volume reconstruction and this reference (Fig. 2g) showed that PHYSIQ nearly doubled SSIM relative to the raw input and approached the performance of the 10× averaged reference, whereas BM3D yielded only modest improvement and N2V showed negligible gain. We further quantified image quality using an LD-mask-based signal-to-noise ratio (SNR), defined as the mean LD intensity within 3D LD masks divided by the standard deviation of an equivalently processed background region. Under this LD-centered metric, PHYSIQ achieved a 12.5 dB SNR improvement over the raw input across fields of view (Fig. 2h), consistent with gains reported for state-of-the-art self-supervised denoising approaches (typically 10–12 dB)^33^.

To avoid bias introduced by temporally averaged cellular references, we further established a motion-free static phantom benchmark using 3 μm polystyrene-poly(methyl methacrylate) (PS/PMMA) microspheres (Fig. 2i-k). A low-SNR bead volume acquired under 5 mW excitation (Fig. 2i) was reconstructed using PHYSIQ (Fig. 2j) and compared against a high-SNR reference image (Fig. 2k) obtained from the same field of view using identical excitation power combined with 60-frame temporal averaging. This static reference eliminates biological motion and prevents temporal blurring or positional averaging artifacts. As shown in Fig. 2l, PHYSIQ restores bead morphology with high fidelity, closely matching the long-integration reference and substantially improving structural similarity (SSIM = 0.95) relative to the raw input (SSIM = 0.54). The speed-SNR analysis (Fig. 2m) further demonstrates that PHYSIQ achieves substantial SNR enhancement from a single-volume acquisition, whereas temporal averaging improves SNR only at the expense of temporal resolution.

To further evaluate spatial-frequency fidelity beyond conventional SNR and SSIM metrics, we performed paired Fourier ring correlation (FRC) analysis on HeLa cell data before and after denoising (Supplementary Note S5 and Fig. S3). PHYSIQ increases reproducible spatial-frequency content in the intermediate-frequency range without introducing artificial high-frequency components, indicating that the reconstruction preserves intrinsic cellular structure without detectable hallucination under the tested conditions.

From a physical perspective, the present implementation operates within fundamental measurement constraints. Fast volumetric SRS imaging is inherently SNR-limited, where precision in estimating Raman contrast is bounded by the Fisher information and the corresponding Cramér-Rao lower bound^40–42^. Accordingly, PHYSIQ does not seek to surpass the shot-noise limit. Instead, it provides a physically grounded strategy to generate independent noisy measurements of the same latent Raman signal^43^, enabling self-supervised restoration that approaches the effective information content of rapid low-SNR acquisitions without requiring temporal averaging.

### PHYSIQ enables label-free volumetric motility phenotyping of lipid droplets

**Fig. 3:**
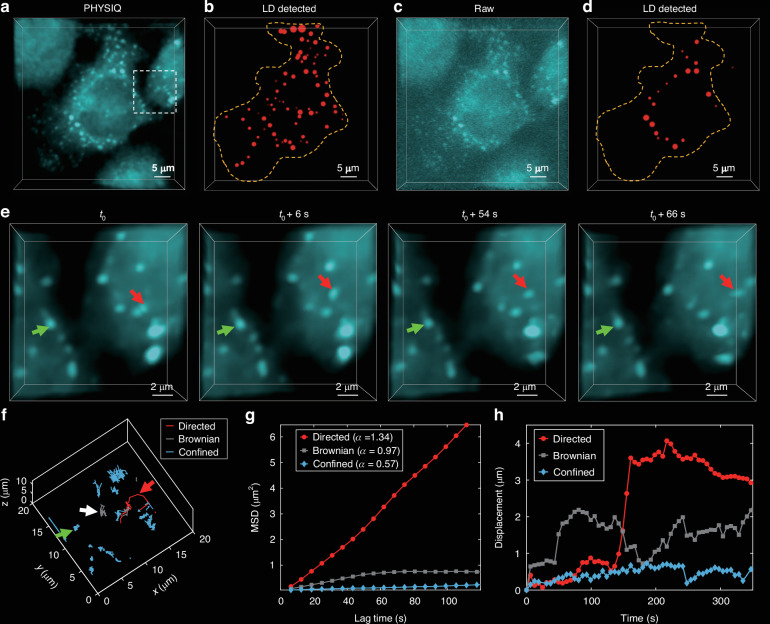
Volumetric lipid droplet (LD) kinematics enabled by PHYSIQ. **a**, **c** Comparison of PHYSIQ-reconstructed (**a**) and raw (**c**) SRS volumes, showing enhanced LD contrast and structural clarity with PHYSIQ. **b**, **d** 3D LD detection maps (red dots) within the cell boundary (yellow dashed line) reveal dense LD identification using PHYSIQ (**b**) but sparse detection in raw data (**d**). **e** Time-lapse volumetric views of the boxed region in (**a**) showing representative LD motions: directed (red arrow), Brownian-like (white arrow), and confined (green arrow). **f** Corresponding 3D trajectories color-coded by motion class: directed (red), Brownian-like (gray), and confined (blue). **g** Mean squared displacement (MSD) curves and (**h**) cumulative displacement traces for the representative trajectories in (**f**). Scale bars: 5 µm (**a**–**d**), 2 µm (**e**). Imaging conditions: 320 × 320 × 8 voxels; pixel dwell time, 4 µs; paired I/Q 2D frame time, 775 ms; 3D volume time, 6.2 s

We next applied PHYSIQ to track LDs in three dimensions within live cells using the 2850 *cm⁻¹* Raman band, which is specific to cellular lipids (See Supplementary Video 2). Figure 3 demonstrates that PHYSIQ markedly enhances LD detectability compared to raw SRS data. Following PHYSIQ reconstruction (Fig. 3a), LDs appear as compact, high-contrast features, enabling dense and consistent mapping throughout the entire cell volume (Fig. 3b; dashed outline denotes the cell boundary). In contrast, raw images exhibit a blurred and noisy background in which many LDs are obscured (Fig. 3c). As a result, conventional SRS analysis under low-SNR conditions yields sparse and unreliable LD detection (Fig. 3d). These improvements directly strengthen frame-to-frame identity assignment and enable robust 3D tracking of individual LDs over time.

We then extended analysis to full 4D (3D + time) tracking to resolve intracellular motion heterogeneity. In Fig. 3e, time-lapse zoom-ins retain high contrast across all frames, enabling unambiguous tracking of individual LDs. Distinct motility behaviors are evident: some LDs undergo persistent long-range transport (red arrows), whereas others remain spatially confined within localized regions (green arrows). The resulting 3D trajectory map (Fig. 3f) reveals pronounced intracellular heterogeneity, including long, directed runs spanning several micrometers, near-isotropic random walks consistent with Brownian-like motion, and strongly confined trajectories. To quantitatively characterize LD dynamics, we computed the mean-squared displacement (MSD) for individual trajectories^44,45^ and classified motion states by fitting MSD to a power-law relation, MSD(τ) ∝ τ^α^ (see “Methods”), where the scaling exponent α distinguishes directed, diffusive, and confined regimes. As shown in Fig. 3g, representative MSD curves exhibit distinct behaviors: directed trajectories show super-linear growth, Brownian-like motion approaches linear scaling, and confined motion displays early saturation. The corresponding fitted exponents were *α* = 1.34 for directed motion, *α* = 0.97 for Brownian-like motion, and *α* = 0.57 for confined motion. Cumulative displacement traces (Fig. 3h) further corroborate these classifications, showing sustained growth for directed transport, fluctuating intermediate displacement for diffusive motion, and rapid plateauing for confined droplets. The above results demonstrate that PHYSIQ enables robust label-free 3D single-cell tracking of LDs and quantitative extraction of volumetric kinematic signatures in living cells.

To further investigate whether kinematically directed LD trajectories are associated with cytoskeleton-dependent transport, we performed microtubule perturbation experiments using nocodazole. Following 1 h of nocodazole treatment, LD trajectories became markedly shorter and less persistent compared with untreated controls, exhibiting reduced total displacement and decreased MSD amplitudes (Supplementary Note S6 and Fig. S4). In parallel, the proportion of directed trajectories decreased, while confined and restricted motion states became more prevalent. These observations indicate that the long-range, directionally biased LD motions resolved by PHYSIQ are sensitive to microtubule disruption, consistent with a microtubule-dependent contribution to directed transport, while preserving the validity of the three identified kinematic states.

### Lipid loading reprograms the spatial organization and motility bias of LD networks

**Fig. 4:**
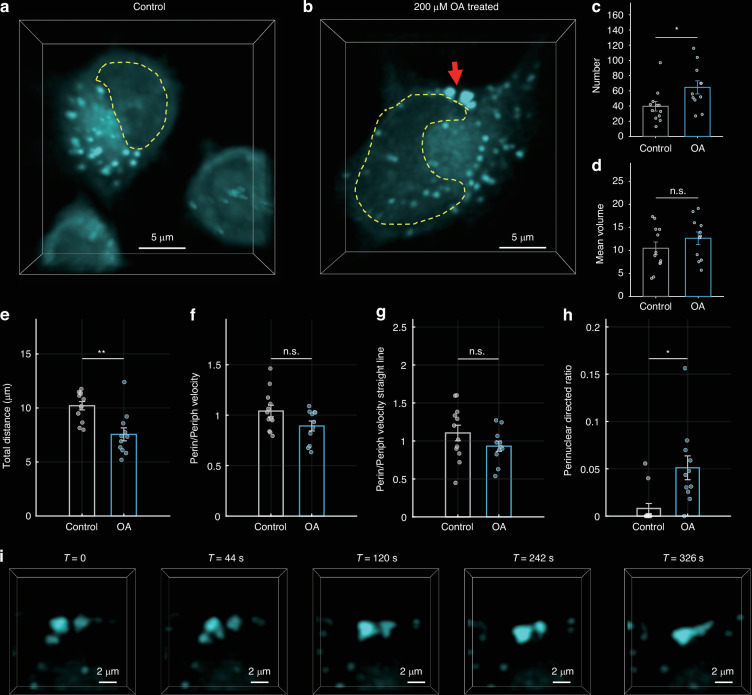
Nutrient-induced spatial redistribution and kinematic remodeling of lipid droplets (LDs). **a**, **b** Representative 3D renderings of LDs in HeLa cells under control (**a**) and lipid-loaded (200 µM oleic acid, OA) conditions (**b**). Yellow dashed lines mark the perinuclear region. **c** Number of tracked LDs per cell. **d** Mean LD volume across whole cells. **e** Total trajectory distance of LD motion. **f** Perinuclear-to-peripheral LD velocity ratio. **g** Perinuclear-to-peripheral straight-line velocity ratio, reflecting transport efficiency. **h** Fraction of directed LD motion in the perinuclear region. **i** Time-lapse 3D sequence showing dynamic interaction and progressive LD contact of peripheral LDs in an OA-treated cell over 326 s (red arrow in (**b**) indicates tracked LDs). For **e**–**h**, each dot represents one independently imaged cell-level trajectory summary. *n* = 12 for control and *n* = 11 for OA-treated cell datasets. Data acquired from >3 independent biological replicates. Data are mean ± SEM with individual data points representing single cells. Two-group comparisons were performed using two-sided Welch’s t-tests. For the predefined primary kinematic endpoints in **e**–**h**, *p* values were adjusted using the Holm-Bonferroni method; significance labels are based on Holm-adjusted *p* values: * Holm-adjusted *p* < 0.05, ** adjusted *p* < 0.01; n.s., not significant. Imaging conditions: 256 × 256 × 8 voxels; pixel dwell time, 4 µs; paired I/Q 2D frame time, 550 ms; 3D volume time, 4.4 s

To investigate how lipid loading reshapes intracellular LD organization and trafficking, we applied the PHYSIQ platform to compare 3D LD dynamics in control HeLa cells and cells treated with 200 μM oleic acid (OA). Figure 4a, b show reconstructed volumetric images revealing pronounced alterations in both LD abundance and subcellular distribution. Under control conditions, LDs are relatively sparse and exhibit a preferential enrichment in the perinuclear cytoplasm (Fig. 4a). In contrast, OA-treated cells display larger, more intensely scattering droplets that are distributed more broadly throughout the cytoplasm (Fig. 4b). The lipid-poor nucleus appears as a distinct low-intensity region in the 2850 *cm⁻¹* channel^46^, enabling nuclear boundaries to be delineated based on this negative contrast (Fig. 4a, b, dashed outlines). Quantitative analysis (Fig. 4c, d) shows that OA treatment increases both the number of detected LDs per cell and the mean LD volume, consistent with concurrent LD biogenesis and growth under lipid-rich conditions^47^. At the single-cell level, OA treatment significantly reduces the total trajectory displacement of LDs (Fig. 4e; Holm-adjusted *p* = 0.0070, Hedges’ *g* = 1.51), indicating that lipid-loaded cells exhibit a more spatially constrained mode of LD trafficking.

Next, we studied how LD movement changes based on their location in the cell. We defined a relative position metric, $\rho ={d}_{n}/({d}_{n}+{d}_{m})$, where *d*_*n*_ and *d*_*m*_ represent the Euclidean distances to the segmented nuclear and cell membranes, respectively. LDs were divided into perinuclear ($\rho < 0.3$) and peripheral ($\rho > 0.7$) groups to quantify compartment-specific trajectory analysis. The changes of LDs movement were not uniform across the cell. The perinuclear-to-peripheral velocity ratio was decreased in OA-treated cells (Fig. 4f, Holm-adjusted *p* = 0.131), and a similar reduction was observed for the perinuclear-to-peripheral velocity straight line (Fig. 4g, Holm-adjusted *p* = 0.174) which reflects the efficiency of effective movement from the starting point to the destination. These results suggest that lipid loading tends to reduce the motility of perinuclear LDs relative to those located in the cell periphery. Notably, despite this reduction in net movement near the nucleus, the percentage of directed trajectories within the perinuclear compartment increased significantly following OA treatment (Fig. 4h, Holm-adjusted *p* = 0.023, Hedges’ *g* = −1.30), suggesting OA-induced lipid loading imposes stronger local crowding or confinement near the nuclear zone while simultaneously promoting a subset of directionally biased LD motions. Consistent with this interpretation, time-lapse volumetric zoom-ins revealed progressive LD contact and shape changing over time (Fig. 4i), indicating enhanced inter-droplet interactions during OA-driven lipid accumulation (See Supplementary Video 3). These results show that OA treatment does not merely increase LD abundance, but reconfigures the LD network into a more spatially heterogeneous and dynamically biased state, confirming the ability of PHYSIQ to resolve metabolically induced remodeling of intracellular organelle dynamics.

### Glycolytic inhibition suppresses long-range lipid droplet transport

**Fig. 5:**
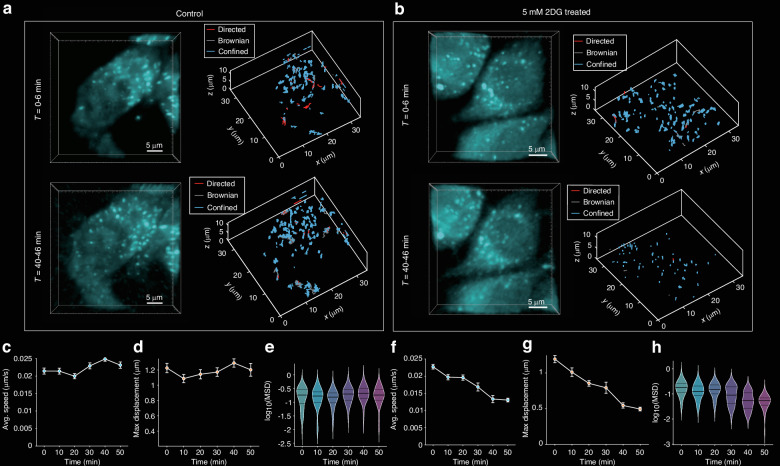
Glycolytic inhibition suppresses lipid droplet (LD) motility. **a** 3D images and trajectory maps from a control cell at early (0–6 min) and late (40–46 min) time points, showing stable LD dynamics with mixed motion types: directed (red), Brownian-like (gray), and confined (blue). **b** Corresponding maps from a cell treated with 5 mM 2-deoxy-D-glucose (2DG), where LD motion becomes predominantly short and confined at late times (40–46 min). Time evolution of average LD speed (**c**) and maximum displacement per track (**d**) in control cells, indicating minimal drift during imaging. **e** Violin plots of log₁₀(MSD) across time bins in control cells. Time evolution of average speed (**f**) and maximum displacement (**g**) following 2DG treatment. **h** Violin plots of log₁₀(MSD) over time after 2DG addition, showing a progressive shift toward smaller step amplitudes. Scale bars, 5 μm. The control group included 241 tracks, while the 2DG-treated group included 170 tracks. Track numbers indicate descriptive trajectory counts from representative single-cell time-course datasets. Imaging conditions: 256 × 256 × 8 voxels; pixel dwell time, 4 µs; paired I/Q 2D frame time, 550 ms; 3D volume time, 4.4 s

To examine whether LD motility is coupled to cellular metabolic activity, we inhibited glycolysis using 2-deoxy-D-glucose (2DG), a competitive inhibitor of hexokinase^48^. In untreated control cells, LD motion remained stable over the full imaging period. Figure 5a shows 3D reconstructions and corresponding tracking maps from an early window (0–6 min) and a late window (40–46 min), revealing a persistent mixture of confined, Brownian-like, and directed trajectories without appreciable temporal drift. Consistently, the mean speed (Fig. 5c) and maximum displacement per trajectory (Fig. 5d) remained largely constant over time. The MSD distributions (Fig. 5e) likewise maintained similar profiles across successive time bins, indicating stable LD step sizes and sustained transport dynamics during prolonged imaging.

In contrast, 2DG treatment induced a progressive, time-dependent suppression of LD motility (Fig. 5b). At early time points (0–6 min), LDs still exhibited mixed motion states; however, at later time points (40–46 min), tracking maps revealed a marked shift toward short, predominantly confined trajectories, accompanied by a reduction in both Brownian-like and directed transport events. Quantitatively, LD speed decreased steadily following 2DG exposure (Fig. 5f), together with an approximately 50% reduction in maximum displacement relative to baseline (Fig. 5g). These trends were further reflected in a progressive downward shift of MSD distributions over time (Fig. 5h), indicating reduced step sizes and suppression of long-range transport.

To validate these observations at the population level, we performed multicell endpoint analysis after 1 h of 2DG treatment (Supplementary Note S7; Fig. S5), which confirmed significantly reduced total LD displacement and decreased MSD scaling exponents, along with a shift in motion-state composition toward confined trajectories. Metabolic inhibition was further verified using two-photon fluorescence imaging (Supplementary Note S8; Fig. S6), which showed an increased optical redox ratio (FAD/[FAD + NAD(P)H]) following 2DG treatment^49,50^, consistent with altered cellular energy balance. All these results indicate that glycolytic inhibition rapidly suppresses LD motility in living cells, supporting a close coupling between LD transport dynamics and cellular metabolic state rather than passive fluctuations.

### Cell division alters the 3D organization and dynamics of lipid droplets

**Fig. 6:**
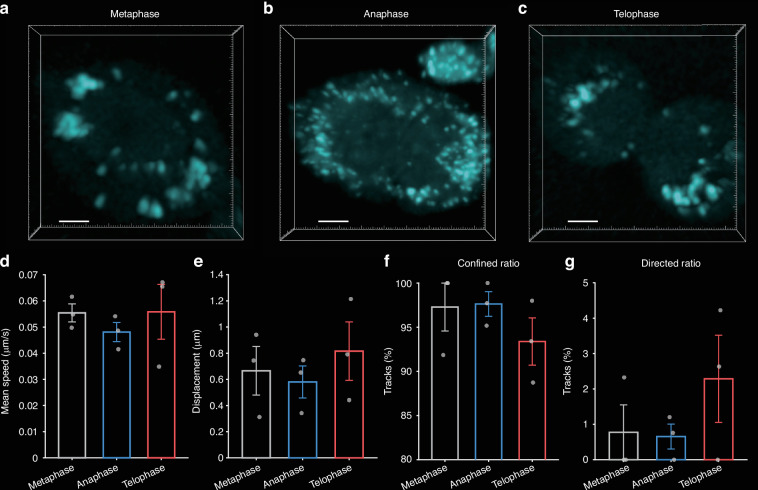
Mitotic phase–dependent redistribution and kinematic dynamics of lipid droplets (LDs). **a**–**c** Representative PHYSIQ-reconstructed 3D LD volumes during metaphase (**a**), anaphase (**b**), and telophase (**c**), showing phase-dependent spatial redistribution across the cell. Population statistics of LD trajectory kinematics across mitotic phases: **d** mean speed and **e** total displacement per track. Motion-mode composition across phases, quantified as the fraction of confined trajectories (**f**) and directed trajectories (**g**). Scale bars, 5 μm. Each dot represents one independently imaged mitotic cell. *n* = 3 cells for each mitotic phase. Data are shown as mean ± SEM. Imaging conditions: 320 × 320 × 8 voxels; pixel dwell time, 4 µs; paired I/Q 2D frame time, 775 ms; 3D volume time, 6.2 s

To investigate how mitotic cellular reorganization influences organelle dynamics, we applied the PHYSIQ platform to perform high-resolution 3D tracking of LDs across distinct stages of cell division. Figure 6a–c reveal pronounced phase-dependent remodeling of LD spatial organization. During metaphase, LDs exhibit strong peripheral enrichment with a central depletion zone (Fig. 6a), consistent with occupation of the cell center by the mitotic spindle^51^. In anaphase, as the cell elongates and chromosomes segregate, LDs redistribute along the extended cell axis, forming a more continuous peripheral distribution (Fig. 6b). In telophase, LDs partition into the emerging daughter cells and begin to re-establish localized intracellular clusters (Fig. 6c).

We next quantified population-level kinematics from reconstructed 3D trajectories to define stage-specific motion signatures. The mean instantaneous speed remained largely invariant across all mitotic stages (Fig. 6d). In contrast, displacement metrics exhibited clear stage dependence, reaching a minimum during anaphase (0.58 ± 0.12 μm) compared with metaphase (0.67 ± 0.19 μm) and telophase (0.82 ± 0.22 μm) (Fig. 6e), indicating that long-range LD excursions are selectively constrained during anaphase, when cytoplasmic geometry and mechanical stiffness are most restricted^52^. Consistently, trajectory classification revealed a dominance of confined motion across all stages (>90%), with confinement peaking during anaphase (Fig. 6f), further indicating maximal suppression of exploratory dynamics at this stage.

Notably, we observed a stage-specific re-emergence of directed transport during telophase. The directed motion fraction, which reached its minimum in anaphase (0.65 ± 0.35%), increased nearly 4-fold to 2.29 ± 1.23% in telophase (Fig. 6g). This late-mitotic increase in directed trajectories is consistent with microtubule-mediated redistribution of LDs into daughter cells as part of a regulated organelle partitioning program^53^. The above results indicate that the cell cycle acts as a temporal regulator of organelle dynamics, coordinating LD redistribution during division to ensure faithful inheritance of metabolic organelles by daughter cells.

Beyond HeLa cells, which are characterized by dysregulated lipid metabolism, we further validated PHYSIQ in live RAW264.7 macrophage-like cells, taking advantage of their distinct lipid-droplet physiology to demonstrate applicability across a biologically different cellular system (Supplementary Note S9 and Fig. S7). PHYSIQ enabled clear, label-free visualization of lipid droplets and supported volumetric tracking of their dynamic behavior in these immune cells. The resulting trajectories revealed heterogeneous motility patterns, including both confined and long-range motion, indicating that the PHYSIQ framework generalizes beyond HeLa cells and is applicable across diverse cellular contexts.

While single-cell tracking reveals phase-dependent organelle kinematics, long-term volumetric imaging in living organisms remains challenging for conventional SRS due to phototoxicity and photodamage. By distributing excitation energy into temporally separated in-phase and quadrature pulses, PHYSIQ reduces peak power relative to conventional SRS (see “Materials and methods”), thereby mitigating photodamage constraints. As a qualitative proof-of-principle demonstration of extended label-free volumetric imaging in a developing organism, we performed continuous PHYSIQ-based SRS 3D imaging of a zebrafish embryo over 70 min. The resulting time-lapse recording captured ongoing developmental dynamics and successive cell division events within the field of view (Supplementary Video 4), illustrating the feasibility of prolonged volumetric SRS observation in live embryonic systems.

## Discussion

Rapid label-free volumetric SRS imaging in living cells is fundamentally constrained by shot noise, particularly under the short pixel dwell times required for fast 3D acquisition, limiting reliable visualization and tracking of dynamic organelles such as LDs^16,54,55^. Conventional solutions, including temporal averaging or prolonged pixel dwell time can improve SNR only by sacrificing temporal resolution and often introducing motion blur or loss of structural fidelity. Existing computational denoisers, including BM3D^26^ and Noise2Void^29^, provide useful benchmarks but rely on assumptions that become unreliable in rapidly evolving intracellular environments, while frame-splitting or repeated-acquisition self-supervised approaches are inherently challenged by dynamic samples^35,56^. PHYSIQ overcomes these limitations through a physics-paired in-phase/quadrature SRS acquisition strategy that generates simultaneous measurements with identical underlying signal but statistically independent shot noise. This enables fully self-supervised Noise2Noise restoration without clean references or repeated imaging, improving effective SNR by ~12.5 dB while preserving fine spatial detail and temporal fidelity (Fig. 2 and Supplementary Note S5).

Beyond its denoising capability, PHYSIQ provides a label-free framework for quantitative 3D organelle phenotyping. In contrast to single-plane imaging, which cannot resolve axial motion and often introduces projection ambiguities^17^, PHYSIQ reconstructs full volumetric lipid-droplet trajectories, enabling direct characterization of LD motility as structured kinematic state spaces comprising directed, Brownian-like, and confined transport modes (Fig. 3a–f). This volumetric perspective preserves critical geometric information required to distinguish long-range displacement, local confinement, and compartment-dependent transport biases that are difficult to infer from 2D projections. These findings extend previous 2D SRS/CARS studies of lipid-droplet dynamics by uncovering the 3D transport architecture underlying intracellular lipid trafficking^17^. More broadly, resolving LD 3D motility states supports the emerging perspective that dynamic intracellular droplet organization can spatially regulate biochemical processes^57,58^.

This capability becomes particularly powerful under metabolic perturbation. During oleic-acid loading, PHYSIQ revealed not only increased LD abundance and redistribution, but also a marked reorganization of transport logic (Fig. 4e-h). Reduced global exploration occurred simultaneously with increased directed trajectories near the nucleus and enhanced LD-LD interactions, including fusion-like remodeling events (Fig. 4i and Supplementary Video 3), indicating spatially heterogeneous trafficking reprogramming rather than a uniform reduction in motility^59^. These findings suggest that lipid loading remodels the spatial logic of intracellular transport, coupling increased local crowding with selective preservation of directional trafficking. By contrast, acute glycolytic inhibition with 2DG rapidly suppressed long-range LD transport and shifted trajectories toward shorter, more confined motion (Fig. 5f-h), while untreated controls remained stable over the same time window. Together with orthogonal optical redox ratio measurements (Supplementary Note S8, Fig. S6), these observations support a strong coupling between LD transport dynamics and cellular metabolic state.

PHYSIQ further enables label-free visualization of dynamic organelle remodeling during cell division. Although fluorescence studies have reported mitosis-associated LD redistribution^60^, continuous volumetric PHYSIQ-SRS imaging resolves both spatial reorganization and motion-state transitions throughout mitotic progression. LDs exhibited maximal confinement during anaphase followed by re-emergence of directed transport during telophase (Fig. 6f,g), indicating that mitosis primarily reshapes accessible transport space and trajectory persistence rather than simply altering instantaneous velocity. These results identify LD kinematic architecture as a dynamic, label-free phenotypic marker of cellular physiological state (Supplementary Note S10 and Fig. S8).

PHYSIQ addresses an important methodological gap between fluorescence microscopy and conventional SRS. Fluorescence-based imaging can perturb LD biology through labeling artifacts, photobleaching, and phototoxicity, whereas conventional SRS offers endogenous chemical specificity but often lacks sufficient sensitivity for rapid volumetric tracking. PHYSIQ bridges this gap by enabling robust 3D tracking of endogenous LDs in living cells without exogenous labels. Although demonstrated here at the CH₂ vibrational band, future hyperspectral implementations could extend PHYSIQ to multiplexed Raman bands, enabling volumetric chemical fingerprinting of organelle composition, lipid heterogeneity, and metabolic remodeling^61,62^. Such capability could support high-throughput 3D metabolic phenotyping in applications ranging from drug response and organoid metabolism to embryogenesis and tumor microenvironment studies.

The physics-paired framework is also extensible beyond same-plane denoising. After model training, PHYSIQ generalized directly to dual-focus volumetric acquisition using axially offset excitation planes, enabling accelerated video-rate 3D imaging without retraining (Supplementary Note S11 and Fig. S9). Further throughput gains may be achieved through resonant scanning or Bessel-beam excitation for deeper and more scattering-tolerant imaging^14,63^. More broadly, PHYSIQ reduces the excitation burden typically required for analyzable volumetric SRS. Conventional approaches often rely on higher laser power, longer dwell time, or repeated averaging (Supplementary Note S12 and Fig. S10), all of which increase optical dose and may exacerbate photothermal stress and phototoxicity during prolonged imaging. By contrast, PHYSIQ improves effective SNR computationally through physics-informed acquisition, enabling reliable extraction of biological information from inherently low-dose measurements. This advantage is supported by long-term continuous zebrafish embryo imaging (Supplementary Video 4). Future integration with quantum-enhanced or squeezed-light SRS^22,64^ could further extend low-dose, high-speed volumetric chemical imaging beyond the classical shot-noise limit.

Importantly, the PHYSIQ concept is modality-agnostic. The same principle, paired measurements with matched signal content but decorrelated noise, can be extended to other laser-scanning systems, including fast two-photon fluorescence imaging of neuronal activity^65^. In such systems, the inter-channel delay used in PHYSIQ remains compatible because fluorescence lifetimes are typically only a few nanoseconds^66^, ensuring complete temporal separation between paired acquisitions.

In summary, PHYSIQ provides a physics-informed route to self-supervised restoration in shot-noise-limited microscopy, transforming rapid volumetric SRS from a sensitivity-limited modality into a robust platform for functional, label-free imaging. By enabling reliable 3D organelle tracking and revealing condition-specific kinematic fingerprints of intracellular dynamics, PHYSIQ opens new opportunities for live-cell biology, metabolic phenotyping, developmental imaging, and biomedical discovery.

## Materials and methods

### PHYSIQ-SRS imaging system (Fig. 1(a))

A dual output ultrafast laser (Insight X3 + , Spectra Physics) operating at a repetition rate of 80 MHz is used as the excitation source in SRS imaging. The fixed output at 1045 nm and a tunable output (680 ~ 1300 nm) serve as the Stokes and pump beams, respectively, for PHYSIQ SRS imaging. To generate two simultaneously recorded SRS images of the same focal volume containing shared Raman signal but independent noise, we implemented a specific spatiotemporal multiplexing scheme. The Stokes beam is intensity-modulated at 20 MHz by an electro-optic modulator (EOM) (APE, GmbH, Berlin) and subsequently split into two distinct optical paths with a temporal offset of 12.5 ns using a polarizing beam splitter (Fig. S1). An additional sub-pulse temporal offset of 5 ps is further applied to suppress residual intra-pulse interference without perturbing the I/Q demodulation. To maintain chemical specificity, linear pulse chirping is applied to both pump and Stokes pulses using SF57 glass rods, equalizing the instantaneous frequencies to match the spectral-focusing bandwidth. A reflective phase-type SLM (Meadowlark 1024 High-speed SLM), positioned conjugate to the objective back pupil, is partitioned into three active zones (Fig. 1(c)): one dedicated to the pump beam and two for the Stokes I/Q paths. Lens phase masks are applied to steer and co-locate the pump focus with the respective Stokes foci within the sample. Residual axial mismatch is corrected by electronically superimposing a defocus phase profile onto the pupil plane of a water immersion microscope objective lens (Apo LWD 25X, NA = 1.05, Olympus). The analytical defocus phase was used as an initial phase estimate. For paired-channel training, residual I/Q lateral and axial misalignments were subsequently corrected using static bead datasets at each axial plane through subpixel image registration. The pump and dual Stokes paths are optically recombined with dichroic mirrors and directed to the sample through a 4-f system composed of a scan lens and a tube lens in the microscope (FV3000, Olympus) to generate twin, co-registered foci. An oil condenser (CC Achromat/Aplanat, NA = 1.4, Nikon) is used to collect the transmitted pump beam, which is isolated from the Stokes beam with a set of bandpass filters (Semrock). A large area photodiode (APE, Berlin) coupled with a phase-sensitive lock-in amplifier (Zurich Instruments, Switzerland) is used to detect the stimulated Raman loss (SRL) signal from the sample for the simultaneous retrieval of the two paired SRS images.

### Imaging parameters

For PHYSIQ-SRS imaging, the average excitation power at the sample was 8 mW for the pump beam and 17 mW for the Stokes beam per focus, within or below the range commonly used for live-cell SRS imaging. For PMMA/PS beads’ motion measurements, the pixel dwell time was set to 0.5 μs, whereas live HeLa-cell imaging was performed with a 4 μs dwell time. Unless otherwise specified, all live-cell datasets were acquired with a lateral pixel size of 133 nm. These imaging conditions were selected to enable PHYSIQ-based volumetric reconstruction under short-dwell, SNR-limited acquisition conditions representative of rapid live-cell imaging.

### PHYSIQ acquisition modes

PHYSIQ was implemented in two operating modes. For self-supervised model training and all live-cell LD imaging experiments, the in-phase (I) and quadrature (Q) channels were axially co-registered and tuned to the same Raman shift. This same-plane physics-paired configuration ensured that both channels contained identical underlying Raman contrast while carrying statistically independent shot-noise realizations, thereby satisfying the requirements for Noise2Noise self-supervised training. For the video-rate microsphere demonstration, an optional dual-foci inference mode was used after PHYSIQ model training. In this configuration, the relative defocus phase encoded on SLM was adjusted to generate two axially separated foci with a 1.5 μm spacing, enabling simultaneous acquisition of two interleaved z-planes. Because the two channels corresponded to different focal planes in this mode, they were not used as paired training inputs; instead, the pretrained PHYSIQ model was applied independently to each channel for inference. Unless otherwise noted, all cellular experiments reported here were performed using the same-plane physics-paired configuration.

### Acquisition timing and volumetric imaging rate

The in-phase and quadrature SRS channels were acquired simultaneously during a single raster scan using dual-phase lock-in detection; therefore, generating a physics-paired I/Q image did not require sequential frame acquisition. For dividing HeLa-cell experiments, each paired 2D frame consisted of 320 × 320 pixels with a measured frame acquisition time of 775 ms. An 8-plane volumetric dataset with a 1.5 μm axial step size therefore required 6.2 s per 3D volume. For oleic-acid-treated and 2-deoxyglucose-treated HeLa cells, each paired frame contained 256 × 256 pixels with a measured frame time of 550 ms, corresponding to 4.4 s per 8-plane volume. Electronic axial scanning was achieved by dynamically updating the defocus phase pattern displayed on the SLM. Because the SLM supports switching rates exceeding 500 Hz (corresponding to <2 ms update time), axial switching overhead was negligible relative to the 2D frame acquisition time and did not limit volumetric imaging speed under the conditions used here.

### SNR quantification

Restoration performance was quantified using an LD mask-based SNR metric. For each LD, a circular lateral mask of fixed radius was defined and extended across all axial slices to generate a corresponding 3D LD region of interest (ROI). For each time point and reconstruction method, the signal term ${I}_{{\rm{LD}}}$ was defined as the mean voxel intensity within the 3D LD ROI. Noise was estimated from a separate background ROI positioned in a signal-free region and propagated through the full z-stack; the noise term ${\sigma }_{{\rm{bg}}}$ was defined as the standard deviation of voxel intensities within this background volume. LD-level SNR was calculated as SNR = 10 log_10_ (I_LD_/σ_bg_). SNR improvement was then quantified relative to the corresponding raw input image.

### Sample preparations

For live-cell imaging experiments, HeLa cells were cultured in DMEM supplemented with high glucose, GlutaMAX, and 10% fetal bovine serum (FBS). Cells were seeded onto glass-bottom dishes and maintained at 37 °C in a humidified incubator with 5% CO₂ for 24 h before imaging. Immediately before SRS imaging, the culture medium was removed and cells were washed twice with phosphate-buffered saline (PBS) at 37 °C and imaged in PBS medium.

### Training and denoising

Prior to network training, paired images were spatially aligned and augmented while preserving the intrinsic noise statistics of the raw measurements, thereby avoiding artificial distortion of the underlying shot-noise patterns. PHYSIQ denoising was implemented using a three-level 3D U-Net architecture optimized for volumetric data, comprising 3 × 3 × 3 convolutional layers with ReLU activation, 2 × 2 × 2 max-pooling layers for down-sampling, and transposed convolution layers for up-sampling, enabling preservation of both spatial and temporal correlations within the volumetric image stack. The network was trained using a cross-validated Noise2Noise framework, in which each physics-paired channel served alternately as input and target. Model optimization used the Adam optimizer with mean squared error (L2) loss and an initial learning rate of 1 × 10⁻⁴. Training was performed using 10,000 normalized 3D patches of size 32 × 32 × 8 voxels with a batch size of 4.

During inference, full volumetric datasets were processed using a sliding-window prediction strategy with 50% overlap and Hanning-weighted patch blending to minimize stitching artifacts at patch boundaries. Final denoised outputs were generated by averaging the predictions from the two cross-trained models, allowing the ensemble to leverage complementary restoration characteristics and further improve signal fidelity. Training was performed on a commercial graphics processing unit (GPU; RTX 3090, NVIDIA) and required ~40 min for model convergence. Inference required ~30 s to denoise a complete volumetric image stack.

For benchmarking, BM3D was included as a representative classical prior-based denoising method, whereas Noise2Void (N2V) was used as a representative self-supervised blind-spot learning approach. These comparisons are particularly relevant for fast raster-scanned SRS imaging, where low-SNR measurements, sparse subcellular structures, and spatially correlated lock-in noise can violate the assumptions underlying both nonlocal patch-redundancy methods and blind-spot prediction strategies, thereby limiting denoising performance.

### Static phantom benchmark

To enable motion-free quantitative evaluation of denoising performance, 3 μm PS/PMMA microspheres were imaged as static phantom structures. Low-SNR bead volumes were acquired under low-power excitation conditions and reconstructed using PHYSIQ from paired I/Q measurements. A high-SNR reference dataset was acquired from the same field of view using 5 mW excitation with 60 repeated acquisitions; these frames were registered when necessary and averaged to generate a long-integration ground truth.

Structural similarity index (SSIM) was computed within bead-centered 3D regions of interest (ROIs) between each reconstructed volume and the corresponding reference. To avoid bias arising from global intensity scaling differences between low- and high-excitation conditions, each ROI was linearly intensity-normalized to the reference prior to SSIM computation. Signal-to-noise ratio (SNR) was evaluated using the original (non-normalized) intensity values, defined as the ratio of the mean bead ROI intensity to the standard deviation of a bead-free background ROI.

### Fourier ring correlation (FRC) analysis

FRC analysis was performed to assess whether PHYSIQ denoising preserves the intrinsic spatial-frequency content of cellular SRS images. For fixed-cell datasets, FRC was computed both between independent raw physics-paired SRS images and between independently reconstructed PHYSIQ outputs. All comparisons were performed on the same axial plane and field of view. When required, image pairs were laterally registered prior to analysis to ensure accurate spatial alignment.

Prior to Fourier transformation, images were mean-subtracted and apodized using a Hann window to suppress boundary artifacts. The Fourier transforms of each image pair were then correlated over concentric spatial-frequency rings to generate the FRC curve. The 1/7 criterion was used as a reference threshold for estimating the effective spatial-frequency support. This analysis was used to evaluate whether PHYSIQ denoising introduces spatial-frequency attenuation or artificial enhancement across the relevant resolution band.

### Trajectory classification based on mean square displacement (MSD)

LD tracking was performed using TrackMate software (Fiji). Single LD trajectories were obtained from time-lapse volumetric imaging and represented as positions $r(t)=[x(t),y(t),z(t)]$ sampled at a constant frame interval ($\Delta t$)^45,67^. For each trajectory of length (N) frames, we computed the time-averaged mean-squared displacement (MSD) at lag time $\tau$ as:

5 $${MSD}\left(\tau \right)={\left\langle |\left|r\left(t+\tau \right)-r\left(t\right)\right|{|}^{2}\right\rangle }_{t}$$

where the average is taken over all valid time pairs (t, t+ $\tau$) within the same trajectory. To focus on reliable short-time dynamics and avoid statistical instability associated with limited sampling at large $\tau$ (where the number of available pairs decreases), all model fitting was restricted to the first four lag times ($\tau \,={k}\Delta t,{k}=1\ldots 4$). A short-time mobility metric was additionally defined as MSD ($\Delta t$) (i.e., the first MSD point). For visualization of MSD curves, each trajectory was plotted only up to $\tau \le T/4$, where $T=(N-1)\Delta t$ is the total duration of the trajectory.

To quantify scaling behaviours, we estimated the anomalous exponent $\alpha$ by linear regression on the logarithmic scale:

6 $${log }_{10}{MSD}\left(\tau \right)=\alpha {log }_{10}\left(\tau \right)+c$$

After logarithmic transformation, a positive linear relationship was modelled using least-squares fitting, with variance explained by the coefficient of determination (r^2^). Only trajectories exhibiting a robust fit (r² > 0.8) were included in the classification. Based on the fit exponent α, trajectories were classified into three distinct motion modes: trajectories with *α* < 0.9 were categorized as confined motion, whereas those with *α* > 1.1 were classified as directed motion, and Brownian motion (0.9 $\le$ *α* $\le$ 1.1), which corresponds to passive, thermally driven random walks^44,62^.

### Statistics and reproducibility

Statistical analyses were performed on independently imaged cell-level summary measurements unless otherwise stated. Individual LD trajectories within the same cell were treated as nested observations and were not used as independent biological replicates for between-condition hypothesis testing. For each cell, trajectory-level measurements were first summarized after pre-specified quality-control criteria, including a minimum trajectory length of 10 frames and a minimum total path length of 0.2 μm. No statistical significance criterion was used for data inclusion, and no outlier exclusion was applied unless explicitly stated. For the OA perturbation analysis, primary kinematic endpoints were predefined before statistical testing, including total trajectory distance, perinuclear-to-peripheral velocity ratio, perinuclear-to-peripheral straight-line velocity ratio, and the fraction of directed trajectories in the perinuclear region^68^. Two-group comparisons were performed using two-sided Welch’s t-tests, which do not assume equal variance between groups. *p* values were adjusted across the predefined primary endpoints using the Holm-Bonferroni method. Significance annotations in the figures are based on Holm-adjusted *p* values unless otherwise stated. Furthermore, to indicate the true magnitude of the biological perturbations, effect sizes were calculated using Hedges’ g, which applies a correction factor suitable for small sample sizes.

## Supplementary information

Supplementary information

Supplementary Video 1

Supplementary Video 2

Supplementary Video 3

Supplementary Video 4

## Data Availability

Data underlying the results presented in this paper are not publicly available at this time but may be obtained from the corresponding author upon reasonable request. The denoising pipeline is based on the original Noise2Noise principle but is adapted to PHYSIQ physics-paired SRS data by using paired I/Q noisy measurements as input-target pairs, anisotropic 3D U-Net training, robust percentile normalization, bidirectional training and sliding-window volumetric inference. The source code used for the core computational analysis is available at https://github.com/linsl16/PhysicsPaired-N2N-SRS.git. The repository includes the MATLAB implementation of the PHYSIQ paired Noise2Noise denoising workflow and representative data. The repository also includes the MATLAB scripts used for MSD analysis, trajectory-level feature extraction and motion-state classification. LD localization and initial trajectory linking were performed in Fiji (TrackMate) using the parameter settings described in the Materials and methods and Supplementary Information.
