# Supplementary material for: Physics-paired stimulated Raman scattering microscopy enables label-free phenotyping of lipid droplets 3D motility in live cells
Source: Light Sci Appl. 2026 Jul 24;15:330. doi: 10.1038/s41377-026-02435-x (PMC13400741; doi:10.1038/s41377-026-02435-x)
Supplement: Supplementary file 1 — Supplementary information [file 41377_2026_2435_MOESM1_ESM.pdf]

Supplementary information for

Physics-paired stimulated Raman scattering microscopy enables label-free phenotyping of lipid droplets 3D motility in live cells

Shulang Lin[1†], Bin He[1†], Chang Liu[1], Rongxuan Li[1], Le Xin[1], Zhiwei Huang[1,2,3,4]*

[1]Optical Bioimaging Laboratory, Department of Biomedical Engineering, College of Design and Engineering, National University of Singapore, Singapore 117576
[2]National University of Singapore (Suzhou) Research Institute, Suzhou, Jiangsu 215123, China
[3]National University of Singapore Guangzhou Research Translation and Innovation Institute, Guangzhou, Guandong 510700, China
[4]NUS Graduate School for Integrative Sciences and Engineering Programme (ISEP), National University of Singapore, Singapore 119077

[†] **The authors contributed equally.**

[*] **Correspondence to:**
Dr. Zhiwei Huang, Optical Bioimaging Laboratory, Department of Biomedical Engineering, College of Design and Engineering, National University of Singapore, 9 Engineering Drive 1, Singapore 117576
Tel: +65- 6516-8856
Fax: +65- 6872-3069
E-mail: biehzw@nus.edu.sg

This SI file includes:
Supplementary Notes S1-12
Supplementary Figures S1-10
Supplementary Videos 1-4

Table of Contents

Supplementary Notes		Page No.
Note. S1	Derivation of Noise Independence in Dual-Channel SRS.	S-3
Note. S2	The PHYSIQ System	S-6
Note. S3	Experimental validation of statistically independent noise between physics-paired channels.	S-8
Note. S4	Implementation and fair tuning of denoising baselines	S-10
Note. S5	Fourier ring correlation validates spatial-frequency fidelity following PHYSIQ denoising	S-11
Note. S6	Microtubule perturbation suppresses long-range LD motility	S-12
Note. S7	Multicell validation of 2DG-induced suppression of LD motility	S-14
Note. S8	Validation of optical redox ratio changes upon 2DG treatment	S-15
Note. S9	Cross-cell-type validation in live macrophages	S-16
Note. S10	Comparison between volumetric 3D tracking and conventional 2D analyses	S-18
Note. S11	Optional dual-foci inference mode for accelerated volumetric imaging	S-20
Note. S12	Power-scaling comparison of raw SRS and PHYSIQ imaging in HeLa cells	S-22
Supplementary Figures		
Fig. S1	Schematic illustration of the PHYSIQ optical configuration used to generate two physically paired stimulated Raman scattering (SRS) measurements through orthogonal lock-in demodulation	S-7
Fig. S2	Experimental validation of noise independence between physics-paired channels	S-9
Fig. S3	FRC-based validation of spatial-frequency fidelity following PHYSIQ denoising	S-11
Fig. S4	Microtubule perturbation suppresses long-range LD motility	S-13
Fig. S5	Multicell validation of lipid-droplet motility suppression following 2DG treatment	S-14
Fig. S6	2DG induces an oxidative shift in HeLa cell redox state	S-15
Fig. S7	PHYSIQ enables volumetric tracking of lipid-droplet dynamics in live macrophages	S-17
Fig. S8	Volumetric tracking reveals axial LD motion and highlights discrepancies between 3D and 2D kinetic analyses	S-19
Fig. S9	Time-lapse volumetric SRS imaging of polymer beads	S-21
Fig. S10	Power-scaling comparison of raw SRS and PHYSIQ in HeLa cells	S-23
Supplementary Videos		
Video. 1	Raw 3D SRS time-lapse imaging of a fixed HeLa cell acquired at 2850 cm^{-1}	S-24
Video. 2	PHYSIQ enables label-free 3D tracking of lipid droplets in live cells	S-25
Video. 3	Oleic acid loading enhances LD interactions and reprograms 3D trafficking dynamics	S-26
Video. 4	Long-term in vivo volumetric SRS imaging of zebrafish development enabled by PHYSIQ	S-27

Supplementary Note S1. Derivation of Noise Independence in Dual-Channel SRS

The performance of self-supervised Noise2Noise denoising critically depends on the statistical independence of noise between the input and target images. In PHYSIQ, this requirement is fulfilled through temporal multiplexing combined with orthogonal lock-in demodulation, which generates two channels with identical structural information but independent noise realizations.

Signal and Noise Model

In stimulated Raman scattering (SRS) microscopy, the weak Raman modulation is extracted from a strong laser background using high-frequency modulation and lock-in detection. The detected signal can be expressed as

$$S(t)=S_{SRS}(t) + n(t) \quad (1)$$

where $S_{\mathrm{SRS}}(t)$ denotes the true structural signal and $n(t)$ represents detector noise.

Under *MHz* modulation conditions, low-frequency laser fluctuations such as $1/f$ noise are strongly suppressed. Consequently, the dominant noise source is photon shot-noise. After subtracting the mean photocurrent, the shot-noise fluctuation is commonly modeled as a zero-mean stochastic process over the detection bandwidth:

$$E[n(t)] = 0 \quad (2)$$

$$R_n(t,t') = E[n(t)n(t')] = \eta\delta(t-t') \quad (3)$$

where $E[\cdot]$ denotes expectation, η is the shot-noise power spectral density determined by the average photon flux, and $\delta(\cdot)$ is the Dirac delta function.

Orthogonal Lock-In Demodulation

A dual-phase lock-in amplifier demodulates the detector signal by projecting it onto two orthogonal reference functions: an in-phase component (SRS_I) and a quadrature component (SRS_Q). The resulting outputs are

$$\mathrm{SRS}_I = \frac{1}{T}\int_0^T [S_{\mathrm{SRS}}(t) + n(t)]\cos(\omega t)\, dt \quad (4)$$

$$\mathrm{SRS}_Q = \frac{1}{T}\int_0^T [S_{\mathrm{SRS}}(t) + n(t)]\sin(\omega t)\, dt \quad (5)$$

where T is the pixel integration time and ω is the modulation frequency.

The corresponding noise terms in the two demodulated channels are therefore

$$\varepsilon_I = \frac{1}{T}\int_0^T n(t)\cos(\omega t)\, dt \quad (6)$$

$$\varepsilon_Q = \frac{1}{T}\int_0^T n(t)\sin(\omega t)\, dt \quad (7)$$

Because the in-phase and quadrature channels correspond to orthogonal demodulation bases, the detected photons contributing to each channel originate from disjoint subsets of the modulated laser pulse train. Both noise terms therefore have zero mean:

$$E[\varepsilon_I] = E[\varepsilon_Q] = 0. \quad (8)$$

Noise Cross-Covariance

To evaluate the statistical relationship between the I and Q channels, we compute the cross-covariance between ε_I and ε_Q. Since both variables are zero mean, the covariance reduces to

$$Cov(\varepsilon_I, \varepsilon_Q) = E[nI \cdot nQ] \quad (9)$$

Substituting the expressions for the two noise terms gives

$$Cov(\varepsilon_I, \varepsilon_Q) = E[\left(\frac{1}{T}\int_0^T n(t)\cos(\omega t)\, dt\right)\left(\frac{1}{T}\int_0^T n(t')\sin(\omega t')\, dt'\right)] \quad (10)$$

Moving the expectation operator inside the integrals yields

$$Cov(\varepsilon_I, \varepsilon_Q) = \frac{1}{T^2}\int_0^T \int_0^T E[n(t)n(t')]\cos(\omega t)\sin(\omega t')\, dt dt' \quad (11)$$

Using the shot-noise autocorrelation model,

$$E[n(t)n(t')] = \eta\delta(t - t') \quad (12)$$

the double integral simplifies to

$$\mathrm{Cov}(\varepsilon_I, \varepsilon_Q) = \frac{\eta}{T^2}\int_0^T \cos(\omega t)\sin(\omega t)\, dt = \frac{\eta}{2\omega T^2}\sin^2(\omega T). \quad (13)$$

In practical SRS systems, the pixel integration time T is much larger than the modulation period $2\pi/\omega$. Under this condition,

$$\mathrm{Cov}(\varepsilon_I, \varepsilon_Q) \approx 0. \quad (14)$$

Thus, the noise realizations in the in-phase and quadrature channels are effectively uncorrelated. PHYSIQ extends quadrature detection concept from parameter estimation to physics-paired SRS image acquisition, where the I/Q channels provide matched Raman signals with decorrelated shot-noise realizations for self-supervised restoration. Previous analyses of quadrature demodulation have shown that the statistical measurement error of noisy optical signals is governed by the signal-to-noise ratio, averaging time and the Cramér–Rao lower bound (CRLB)[1]. Following the establishment of noise independence, we can evaluate the theoretical limits of measurement uncertainty in our SRS system. In a single-pixel measurement, the objective is to estimate the true Raman signal parameter $\theta = S_{SRS}$ from the noisy observation $x = S_{SRS} + \varepsilon$. Assuming the shot-noise fluctuations follow a Gaussian distribution due to the large photon flux in SRS, the likelihood function for the observation is $P(x \mid \theta) \sim \mathcal{N}(\theta, \sigma^2)$, where the noise variance is $\sigma^2 = \eta/(2T)$. The Fisher information $F(\theta)$ quantifies the amount of information the observable carries about the true signal and

$$F(\theta) = -E\left[\frac{\partial^2}{\partial\theta^2} lnP(x|\theta)\right] = \frac{1}{\sigma^2} \quad (15)$$

According to the Cramer-Rao Lower Bound (CRLB), the variance of any unbiased estimator $\hat{\theta}$ cannot be lower than the inverse of the Fisher Information[2]:

$$Var(\hat{\theta}) \geq F(\theta)^{-1} = \sigma^2 \quad (16)$$

Thus, the precision of estimating Raman contrast is fundamentally limited by the shot-noise variance, or equivalently by the information carried by the detected photons. Conventional SRS improves this bound by collecting more photons through higher excitation power, longer pixel dwell time, or repeated-frame averaging, but these strategies increase optical dose, reduce temporal resolution, or introduce motion blur in live-cell volumetric imaging. PHYSIQ does not bypass this fundamental limit or recover information absent from the raw optical measurement. Instead, it generates two physically paired observations of the same Raman signal with independent noise realizations. This pairing provides the statistical condition required for Noise2Noise restoration: signal components are shared between the two channels, whereas stochastic shot-noise components are not. The deep-learning model therefore acts as a regularized estimator that uses spatial context and physics-paired observations to suppress stochastic noise while remaining constrained by the information encoded in the detected photons. In this sense, PHYSIQ improves the practical recoverability of Raman information in SNR-limited measurements rather than exceeding the shot-noise or CRLB-defined measurement limit. This can be seen from the Noise2Noise objective:

$$min\, E[\|f(y_I) - y_Q\|^2] \quad (17)$$

where $y = \theta + \varepsilon$ is the measured value. Since ε_Q is zero-mean and independent of y_I, the cross term vanishes, making the noisy-target objective equivalent to estimating the latent Raman signal up to an additive noise variance independent of the network output.

Implication for Self-Supervised Denoising

The orthogonal demodulation process therefore produces two channels that contain the same underlying structural signal but statistically uncorrelated shot-noise realizations. This property satisfies the independence assumption required for Noise2Noise training and enables self-supervised denoising without introducing correlated noise artifacts between training pairs.

Supplementary Note S2. The PHYSIQ System

Figure S1 shows the schematic of the PHYSIQ system used to generate two physically paired SRS measurements through orthogonal lock-in demodulation. In PHYSIQ setup, a mode-locked dual-output femtosecond laser operating at an 80 *MHz* repetition rate provides synchronized excitation beams at 800 *nm* and 1045 *nm*, serving as the pump and Stokes fields for SRS, respectively. The Stokes beam is intensity-modulated at 20 *MHz* using an electro-optic modulator (EOM), corresponding to a modulation period of 50 *ns*. Following modulation, the Stokes beam is directed into a polarization-based beam-splitting stage that generates two separate branches corresponding to the in-phase (I) and quadrature (Q) channels.

To produce orthogonal lock-in phases while maintaining identical Raman contrast, one Stokes branch is routed through a free-space optical delay line. A relative delay of 12.5 *ns* is introduced, corresponding to a $\pi/2$ phase shift of the 20 *MHz* modulation reference. This establishes orthogonality between the I and Q channels in the lock-in detection framework. Importantly, the 80 *MHz* laser repetition rate corresponds to a pulse interval of 12.5 *ns*, meaning that the two delayed branches sample distinct subsets of pulses from the same laser pulse train. Consequently, both measurements interrogate the same focal volume and encode the same Raman structural information, while their associated photon shot noise originates from different excitation pulses and is therefore statistically independent. In practice, an additional fine temporal offset of approximately 5 *ps* may be introduced to suppress residual coherent interference between the two branches without affecting the orthogonal I/Q demodulation relationship, since $5\,ps \ll 12.5\,ns$.

The 800 *nm* pump beam is routed through an independent delay-compensation path before being recombined with the two Stokes branches in a reflective spatial light modulator (SLM)-based beam-shaping system. To ensure equivalent spectral-focusing conditions for both channels, matched dispersive chirping is applied to the pump and Stokes beams using high-dispersion SF57 glass rods. This preserves comparable instantaneous frequency sweeps and Raman excitation bandwidths in the I and Q paths, thereby minimizing systematic spectral discrepancies between the paired measurements. As a result, differences between the two acquired images arise predominantly from stochastic shot-noise fluctuations rather than spectral mismatch.

A phase-only reflective SLM is positioned at a plane conjugated to the back pupil of the objective lens and divided into three independently addressable regions. One region controls the pump beam, while the remaining two regions independently modulate the Stokes-I and Stokes-Q branches. Customized phase masks applied to these regions steer the beams to generate two matched pump–Stokes overlap conditions within the specimen. This configuration produces twin diffraction-limited excitation foci corresponding to the I and Q channels while preserving the effective numerical aperture of the optical system. Residual axial misalignment between the paired focal spots can be corrected electronically by introducing a defocus phase term at the pupil plane. Such pupil-plane wavefront control enables channel-specific axial registration and rapid focal adjustment without requiring mechanical displacement of either the objective or the sample.

After phase modulation by the SLM, the pump beam and the two temporally offset Stokes branches are relayed through the scanning optics and focused into the specimen by the microscope objective. The transmitted excitation beams are collected by the condenser and detected using a large-area

photodiode for SRS signal acquisition. Because the two Stokes branches are separated by a quarter-period temporal offset, the detected SRS signal contains two orthogonal modulation components that can be independently recovered using dual-phase lock-in demodulation referenced to the same 20 *MHz* modulation clock.

This optical architecture generates two physically paired and near-simultaneous SRS measurements that share the same underlying Raman contrast while exhibiting approximately independent shot-noise realizations. These paired measurements satisfy the requirements for Noise2Noise self-supervised denoising and enable high-speed volumetric live-cell SRS imaging without the need for clean ground-truth data.

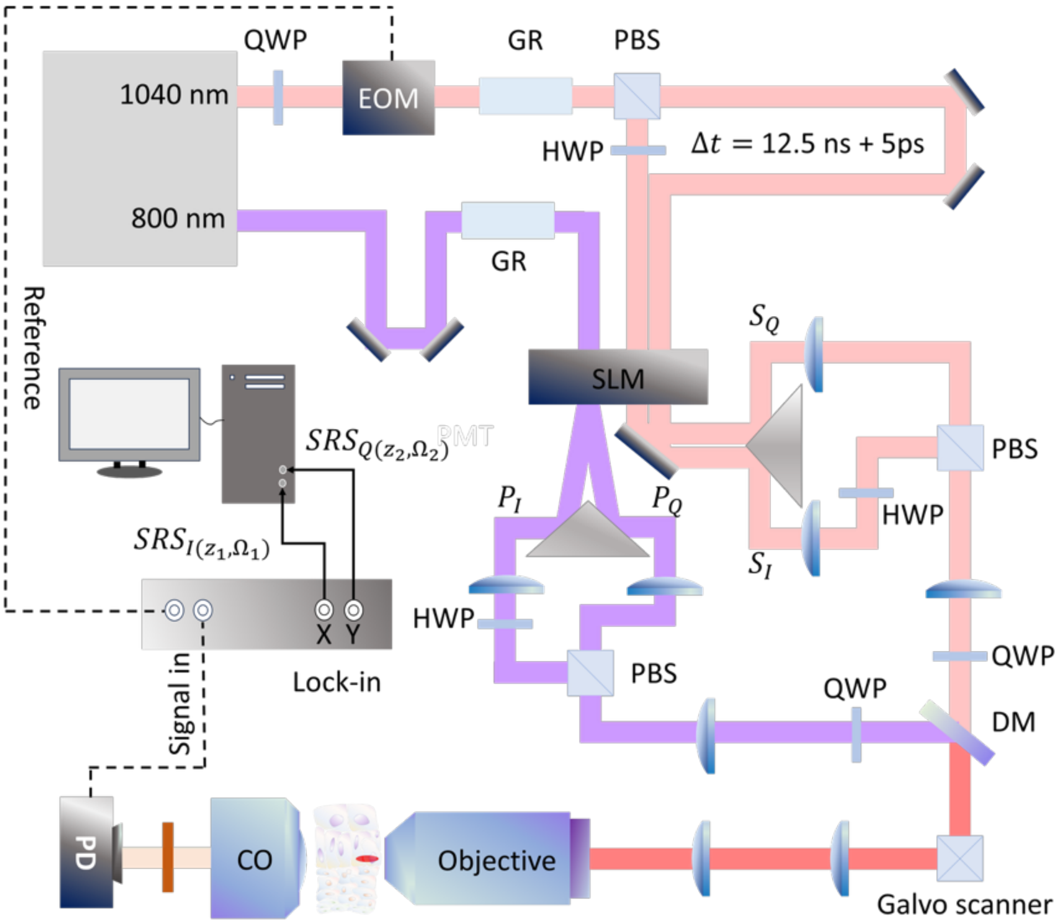

Figure S1. Schematic illustration of the PHYSIQ optical configuration used to generate two physically paired stimulated Raman scattering (SRS) measurements through orthogonal lock-in demodulation.

QWP, quarter-wave plate; EOM, electro-optic modulator; GR, glass rods; PBS, polarizing beam splitter; SLM, spatial light modulator; HWP, half-wave plate; DM, dichroic mirror; CO, condenser; PD, photodiode.

Supplementary Note S3. Experimental validation of statistically independent noise between physics-paired channels

To experimentally confirm that the two physics-paired acquisition channels meet the paired-observation requirement for self-supervised denoising, we evaluated whether they capture identical sample content while exhibiting effectively uncorrelated residual noise. For each axial plane, we generated high-pass residual maps by subtracting a Gaussian-blurred version of the image (σ = 1.0 pixel) to remove shared low-spatial-frequency structure and isolate residual fluctuations primarily arising from detection noise.

Figures S2a and S2b show representative residual maps from the two physics-paired channels, with no observable structured features or shared spatial patterns. The joint distribution of residual intensities pooled across the entire volume (Fig. S2c) is centered around zero and exhibits near-radial symmetry, indicating minimal cross-covariance between channels. To further quantify channel independence, slice-wise Pearson correlation coefficients were calculated from high-pass residual maps at different pixel dwell times (Figs. S2d–f). At a dwell time of 4 μs, residual correlations were effectively zero, with a median absolute correlation of 0.0023 and a maximum of 0.0060 (Fig. S2d). Similar results were obtained at 2 μs, yielding median and maximum absolute correlations of 0.0027 and 0.0098, respectively (Fig. S2e). Even at the shortest dwell time of 0.5 μs, residual correlations remained low, with a median absolute correlation of 0.0156 and a maximum of 0.0323 (Fig. S2f). Together, these results demonstrate that the physics-paired channels retain the same underlying sample information while generating effectively independent noise realizations, thereby satisfying the key assumption required for Noise2Noise-based self-supervised training in PHYSIQ.

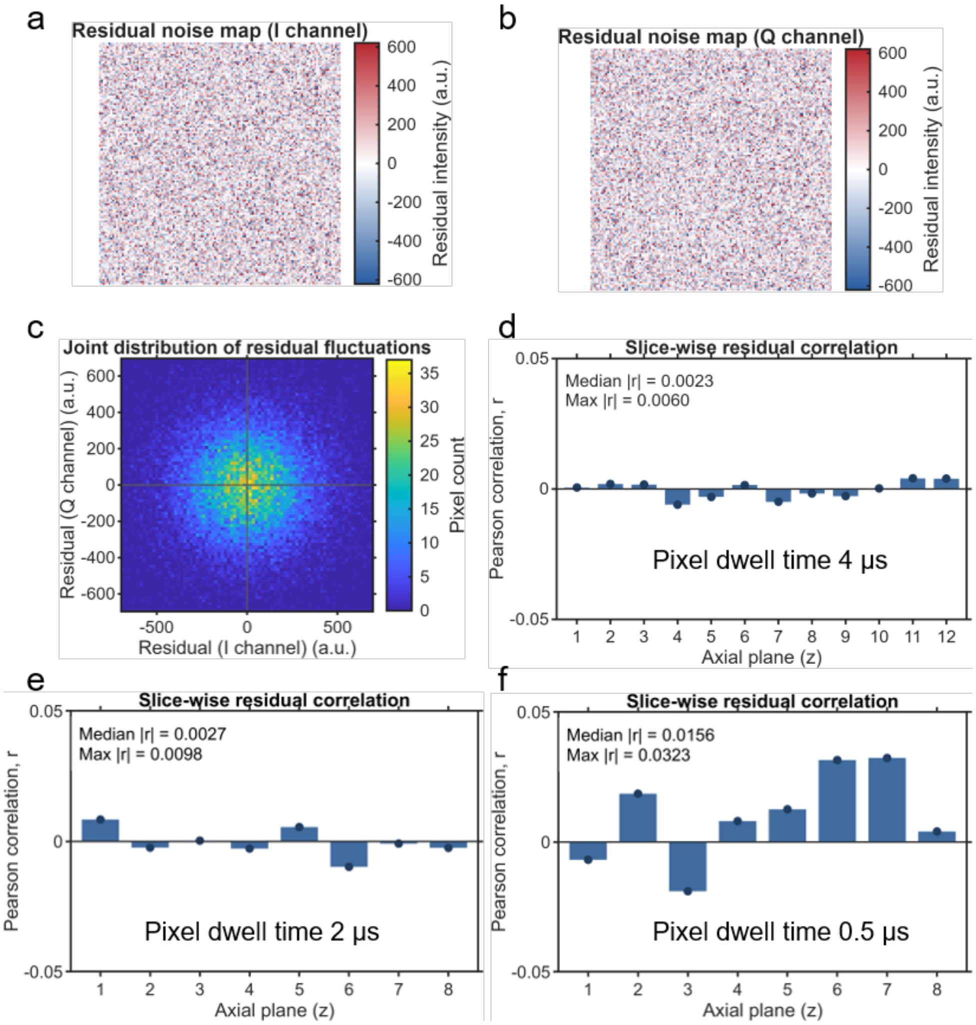

Figure S2. Experimental validation of noise independence between physics-paired channels.

(a) High-pass residual map from the in-phase (I) channel at the central axial plane, obtained by subtracting a Gaussian-blurred version of the raw image (σ = 1.0 pixel) to remove low-spatial-frequency sample features. The I and Q channels were acquired with a 12.5 *ns* temporal offset, ensuring that the underlying sample signal remained effectively unchanged between acquisitions. (b) Corresponding high-pass residual map from the quadrature (Q) channel at the same axial plane, displayed using the identical intensity scale as in (a). Data were acquired with a pixel dwell time of 4 μs. The absence of discernible common structures indicates that the residual fluctuations are dominated by channel-specific noise. (c) Joint distribution of residual intensities pooled from both channels across the imaging volume, with spatial subsampling applied for visualization efficiency. The distribution is centered near zero and exhibits near-radial symmetry without preferential elongation along the diagonal, indicating negligible cross-covariance between channels. (d–f) Slice-wise Pearson correlation coefficients calculated from high-pass residual maps acquired at pixel dwell times of 4 μs, 2 μs, and 0.5 μs, respectively. Residual correlations remained close to zero across all tested conditions, confirming that the physics-paired I/Q channels provide effectively decorrelated observations suitable for Noise2Noise-based self-supervised training.

Supplementary Note S4. Implementation and fair tuning of denoising baselines

To ensure a rigorous and unbiased comparison, the baseline denoising methods (Block-Matching and 3D Filtering (BM3D) and Noise2Void (N2V)) were individually optimized for each dataset rather than relying on default parameters.

BM3D optimization. For the classical BM3D approach, we implemented an automated parameter tuning procedure to avoid suboptimal default settings. Volumetric time-series data were processed slice-by-slice. Before processing the full dataset, a grid search over the noise standard deviation, $\sigma \in \{0.001, 0.005, 0.01, 0.02, 0.05, 0.1\}$, was performed on a representative central slice to identify the σ value that minimizes mean squared error relative to the raw noisy input. To preserve quantitative fidelity, all BM3D operations were performed in double-precision, and the restored outputs were returned to their native dynamic range (e.g., 16-bit) without any min-max contrast normalization.

Noise2Void training and inference. As a self-supervised deep learning baseline, N2V was trained from scratch on each dataset to avoid pre-training biases and accurately model the intrinsic noise characteristics of the microscope. The network employs a lightweight three-level 2D U-Net architecture with 3×3 convolutional layers (ReLU activation), 2×2 max-pooling and transposed convolution layers, and feature channels scaling from 32 to 128 at the bottleneck. To implement the blind-spot self-supervision, 1.5% of pixels in each training patch were masked and replaced by a randomly sampled neighboring pixel within a 5×5 local window.

The network was trained using the Adam optimizer with a masked L1 loss computed exclusively over masked pixels, an initial learning rate of 4×10^{-4}, for 10,000 iterations (50 epochs) on 64×64 normalized 2D patches with a batch size of 16. Data augmentation included random cropping, flipping, and rotation. During inference, full-frame images were processed with a sliding-window scheme (32-pixel overlap, 50%) using 2D Hanning weighting to minimize boundary artifacts. Training was performed on an NVIDIA RTX 3090 GPU, requiring approximately 40 minutes, and inference for an entire image stack took ~20 *s*.

While N2V has shown strong performance in fluorescence microscopy, we observed suboptimal results for fast-scanning SRS data, including noticeable structural blurring. This limitation arises from a fundamental violation of N2V's assumption that noise is spatially uncorrelated, which does not hold in our high-speed SRS acquisition.

Supplementary Note S5. Fourier ring correlation validates spatial-frequency fidelity following PHYSIQ denoising

To assess whether PHYSIQ denoising preserves spatial-frequency information without introducing artificial high-frequency features, we performed Fourier ring correlation (FRC) analysis on SRS images of HeLa cells. FRC was computed between the two independent raw physics-paired channels prior to denoising (Fig. S3a) and between their corresponding independently reconstructed PHYSIQ outputs after denoising (Fig. S3b).

The raw paired images exhibited substantial shot-noise background, leading to rapid decorrelation at higher spatial frequencies. In contrast, PHYSIQ reconstruction markedly suppressed background noise while preserving consistent lipid-rich cellular structures across the paired outputs. As shown in Fig. S3c, the FRC curve after PHYSIQ denoising displayed improved correlation over the intermediate spatial-frequency range relative to the raw data, indicating enhanced recovery of reproducible structural information from noise-limited measurements.

Importantly, the PHYSIQ FRC curve did not exhibit anomalous extension into higher spatial frequencies, which would suggest artificial detail generation. Instead, the preserved FRC profile supports that PHYSIQ improves image fidelity through noise suppression and signal recovery, without detectable hallucination of high-frequency structures under the conditions tested.

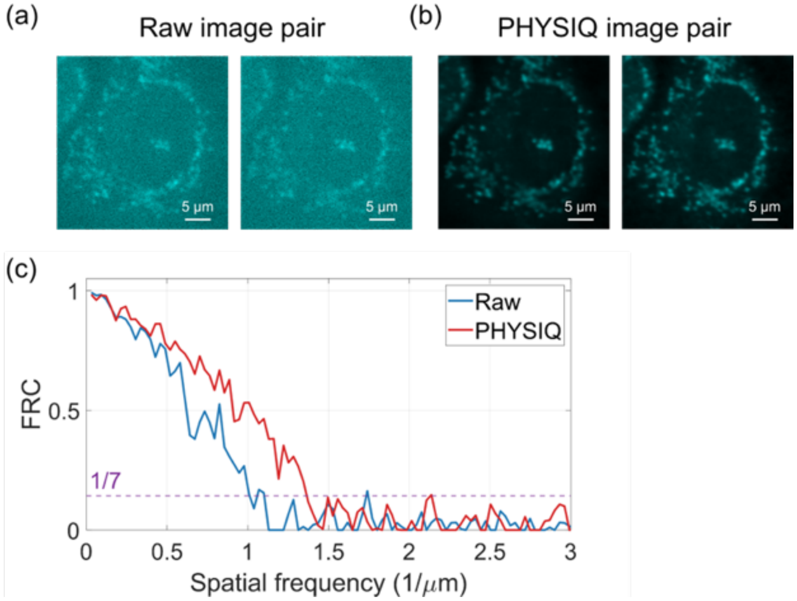

Figure S3. FRC-based validation of spatial-frequency fidelity following PHYSIQ denoising.

(a) HeLa-cell raw paired SRS images acquired from independent channels before denoising. (b) Corresponding independently reconstructed PHYSIQ outputs. (c) FRC curves calculated from the raw paired images and from the paired PHYSIQ reconstructions. The 1/7 threshold is shown as a reference. PHYSIQ increases reproducible structural correlation over the intermediate spatial-frequency range compared with raw data, without abnormal high-frequency extension. This analysis evaluates denoising-related spatial-frequency fidelity rather than absolute optical resolution. Scale bars, 5 μm.

Supplementary Note S6. Microtubule perturbation suppresses long-range LD motility

To assess whether the kinematically directed lipid droplet (LD) trajectories detected by PHYSIQ depend on cytoskeletal transport, we performed microtubule perturbation experiments using nocodazole, a well-characterized microtubule-depolymerizing agent[3] (Fig. S4). HeLa cells were imaged either under untreated conditions or after treatment with 2 μM or 10 μM nocodazole for 1 h, followed by the same PHYSIQ reconstruction, 3D LD tracking, and MSD-based motion-state classification pipeline described in the main text.

As detailed in the main text and Methods, LD trajectories were classified using an MSD-based kinematic criterion: trajectories with scaling exponent $\alpha > 1.1$ were labeled as directed motion, those with $0.9 \leq \alpha \leq 1.1$ as Brownian-like motion, and those with $\alpha < 0.9$ as confined motion. Only trajectories with robust MSD fits were included in the state-composition analysis. The same thresholds and fitting procedure were applied identically across untreated, 2 μM nocodazole, and 10 μM nocodazole conditions. The analysis comprised $n = 5$ independent cells for untreated controls, $n = 5$ for 2 μM nocodazole, and $n = 10$ for 10 μM nocodazole.

Figures S4a–c show representative PHYSIQ-reconstructed LD volumes and 3D trajectories for untreated, 2 μM nocodazole-treated, and 10 μM nocodazole-treated cells, respectively. In untreated cells, a subset of LDs exhibited long, spatially extended trajectories (Fig. S4a). In contrast, nocodazole-treated cells displayed shorter, more locally confined LD tracks (Fig. S4b, c). This reduction in LD exploration is quantified in Fig. S4d, showing that total LD displacement decreased with increasing nocodazole concentration. Consistently, Fig. S4e demonstrates that the MSD scaling exponent from log–log MSD fitting decreased after nocodazole treatment, reflecting reduced trajectory persistence and a shift toward more restricted motion. Motion-state analysis (Fig. S4f) further revealed that the fraction of confined trajectories increased from 84.8% in control cells to 94.2% and 98.0% after 2 μM and 10 μM nocodazole treatment, respectively, with a concomitant reduction in Brownian-like and directed trajectories. These perturbation experiments indicate that the long-range, directionally biased LD motion detected by PHYSIQ is sensitive to microtubule disruption, consistent with microtubule-dependent transport[3].

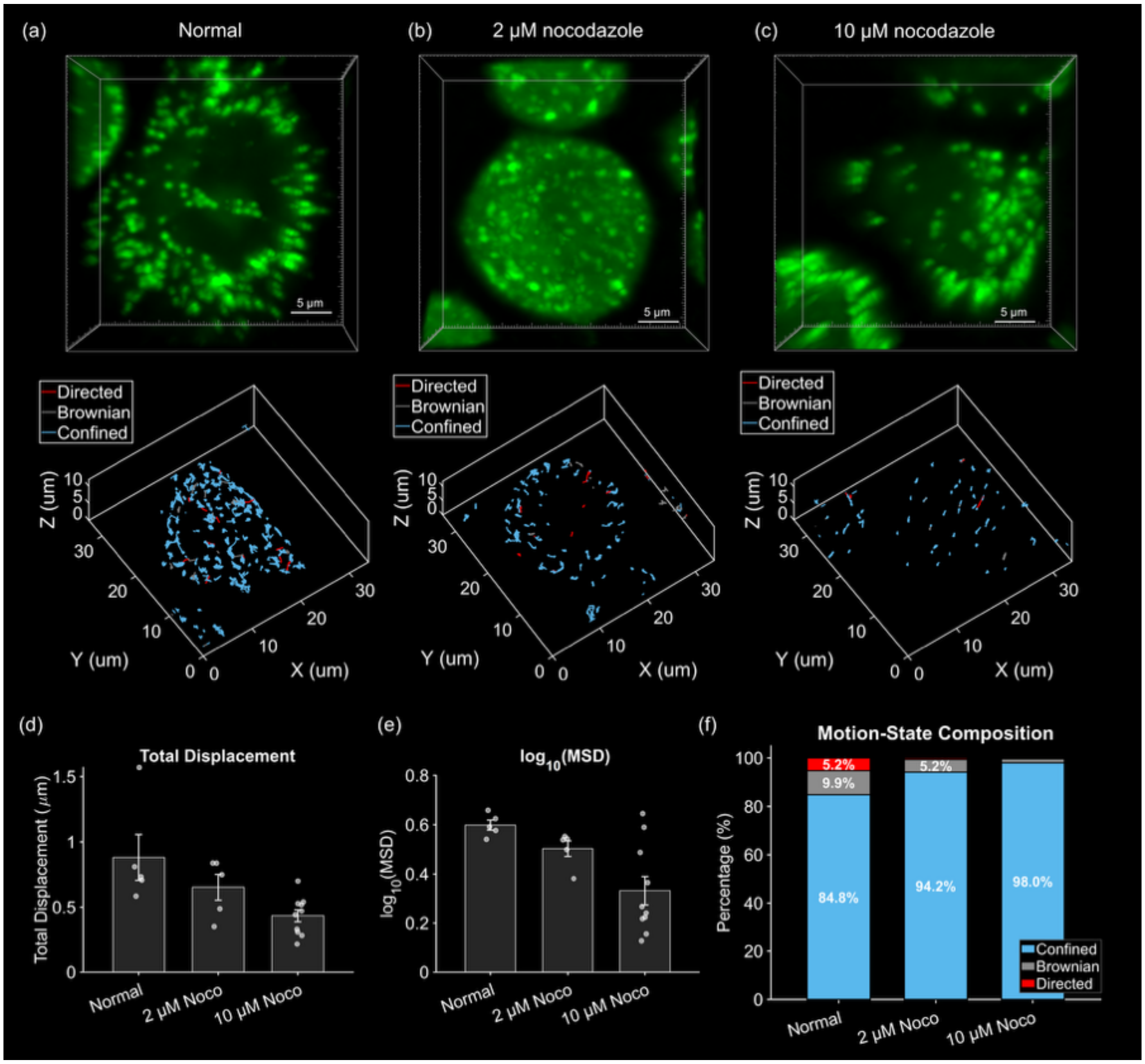

Figure S4. Microtubule perturbation reduces long-range LD motility.

(a–c) Representative PHYSIQ-reconstructed 3D SRS volumes and the corresponding overlaid 3D LD trajectories for (a) untreated control cells, (b) cells treated with 2 μM nocodazole for 1 h, and (c) cells treated with 10 μM nocodazole for 1 h. LD trajectories were classified using MSD-based kinematic criteria (as in the main text) and color-coded as directed, Brownian-like, or confined motion. Nocodazole treatment visibly shortened and spatially confined LD trajectories. (d) Total LD displacement, showing a dose-dependent decrease in LD exploration with increasing nocodazole concentration. (e) MSD scaling exponent derived from log–log MSD fitting, indicating reduced trajectory persistence following microtubule disruption. (f) Motion-state composition for each condition. The fraction of confined trajectories increased from 84.8% in control cells to 94.2% and 98.0% after 2 μM and 10 μM nocodazole treatment, respectively, with corresponding reductions in Brownian-like and directed trajectories. Each data point in panels d and e represents a single cell. Scale bars, 5 μm. Sample sizes: n = 5, 5, and 10 cells for control, 2 μM nocodazole, and 10 μM nocodazole, respectively.

Supplementary Note S7. Multicell validation of 2DG-induced suppression of LD motility

The longitudinal 2-deoxy-D-glucose (2DG) experiment presented in the main text was designed to visualize the temporal evolution of LD motility suppression within the same living cell. To determine whether this phenotype was reproducible across independent biological samples, we performed an endpoint validation experiment after 1 h of 2DG treatment (Fig. S5). To avoid pseudo-replication arising from multiple LD trajectories measured within a single cell, all trajectory-level metrics were first aggregated at the cell level, such that each data point in Fig. S5a, b represents one independently imaged cell rather than an individual LD trajectory.

As shown in Fig. S5a, 2DG treatment significantly reduced the total displacement of LD trajectories relative to untreated controls, indicating diminished intracellular spatial exploration following glycolytic inhibition. Across n = 17 untreated cells and n = 10 2DG-treated cells, LDs in treated cells consistently exhibited shorter cumulative displacements. Consistent with this finding, the MSD scaling slope was significantly decreased after 2DG treatment (Fig. S5b), reflecting a shift away from long-range or directionally persistent transport toward more spatially restricted motion.

Motion-state classification further supported this altered dynamic phenotype. LD trajectories were predominantly classified as confined in both groups; however, the fraction of confined motions increased from 96.2% in untreated cells to 98.7% following 2DG treatment, accompanied by corresponding reductions in Brownian-like and directed motion states (Fig. S5c). Together, these results confirm that acute glycolytic inhibition robustly suppresses LD motility across multiple independent cells, validating the single-cell longitudinal observations reported in the main text.

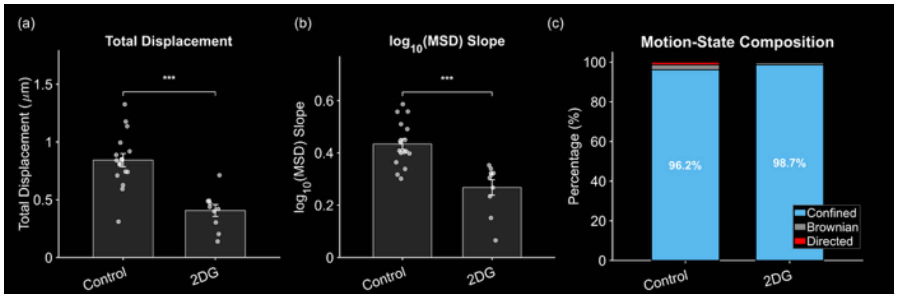

Figure S5. Multicell validation of lipid-droplet motility suppression following 2DG treatment.

(a) Cell-level total displacement of LD trajectories in untreated HeLa cells and cells treated with 2-deoxy-D-glucose (2DG) for 1 h. **(b)** Cell-level short-lag MSD scaling slopes, calculated using the same MSD-based trajectory analysis pipeline described in the main text, showing reduced LD mobility following metabolic inhibition. **(c)** Motion-state composition of LD trajectories in untreated and 2DG-treated cells, classified as confined, Brownian-like, or directed based on MSD scaling behavior; percentages indicate the relative fraction of each motion class. For **(a)** and **(b)**, each data point represents one independently imaged cell, obtained by summarizing all valid LD trajectories within that cell (n = 17 untreated cells; n = 10 2DG-treated cells). Bars indicate mean ± SEM. Statistical significance was assessed using two-sided Welch's t-tests with Holm–Bonferroni correction across predefined endpoint metrics. Total displacement: raw $p = 7.346 \times 10^{-6}$, Holm-adjusted $p = 7.346 \times 10^{-6}$. log10(MSD) slope: raw $p = 2.177 \times 10^{-4}$, Holm-adjusted $p = 2.177 \times 10^{-4}$. ***$p < 0.001$.

Supplementary Note S8. Validation of optical redox ratio changes upon 2DG treatment

To confirm the metabolic perturbation induced by 2DG, we monitored the endogenous autofluorescence of the metabolic coenzymes NAD(P)H and FAD using two-photon microscopy. Cells were excited with a single-wavelength femtosecond laser (800 *nm*), and emitted fluorescence was separated into two detection channels using a dichroic mirror with bandpass filters of 435–485 *nm* for NAD(P)H and 500–550 *nm* for FAD. The optical redox ratio, defined as FAD/[FAD + NAD(P)H], is a widely used label-free metric of intracellular redox state. This normalized ratio ranges from 0 to 1 and reflects the balance between reduced pyridine nucleotides and oxidized flavins. An increase in this ratio typically indicates a shift toward a more oxidized metabolic state, corresponding to decreased NAD(P)H fluorescence[4,5].

As illustrated in Figure S6, 2DG treatment led to an increase in the optical redox ratio in HeLa cells, with pseudo-color maps shifting from predominantly low-ratio blue/cyan regions to higher-ratio yellow/red regions. This effect is consistent with 2DG's established role as a glycolytic inhibitor: once taken up, 2DG is phosphorylated to 2-DG-6-phosphate, which accumulates inside the cell and blocks early steps of glycolysis. By limiting glycolytic flux, the generation of reduced pyridine nucleotides from glucose catabolism is diminished, producing an elevated FAD/[FAD + NAD(P)H] ratio and consequently suppressing overall cellular metabolic activity.

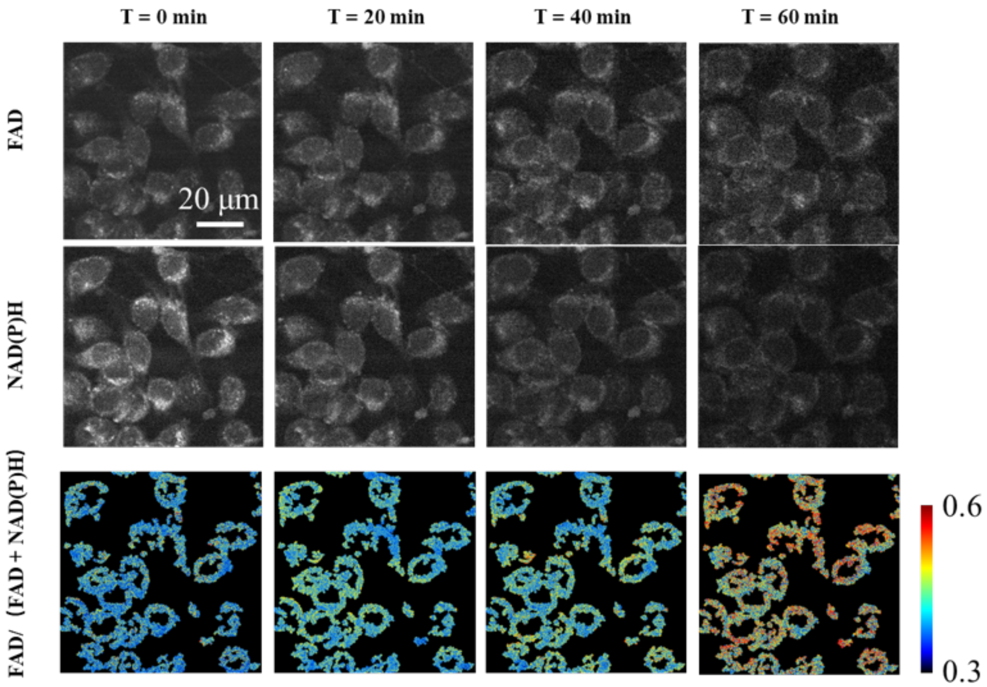

Figure S6. 2DG induces an oxidative shift in HeLa cell redox state.

Representative two-photon autofluorescence images of NAD(P)H and FAD, alongside corresponding pixel-wise optical redox maps computed as FAD/[FAD + NAD(P)H]. Following 2DG treatment, the redox maps exhibit a shift toward higher values, indicating a more oxidized cellular metabolic state consistent with glycolytic inhibition. Warmer colors represent higher optical redox ratios. Scale bar, 20 μm.

Supplementary Note S9. Cross-cell-type validation in live macrophages

To evaluate the generalizability of PHYSIQ beyond HeLa cells, we applied the method to live macrophages, a biologically distinct cell type characterized by markedly different LD organization and trafficking behavior. As shown in Fig. S7a–d, PHYSIQ enabled clear label-free visualization of macrophage LDs compared with the corresponding raw SRS images (Fig. S7a) and resolved their dynamic intracellular redistribution over time (Fig. S7b–d).

Representative three-dimensional LD trajectories reconstructed from the denoised volumetric data revealed heterogeneous motion behaviors, including both spatially confined displacements and pronounced long-range directed transport (Fig. S7e). In this representative macrophage, motion-state classification identified a substantial fraction of directed trajectories, consistent with active long-range LD transport captured by PHYSIQ (Fig. S7f). In Fig. S7g, corresponding MSD curves further distinguished confined, Brownian-like, and directed trajectory classes based on their characteristic scaling behavior.

To assess reproducibility across multiple cells, we extended the analysis to a gallery of OA-treated macrophages (Fig. S7h). Population-level quantification demonstrated that although the relative proportions varied modestly between cells, confined motion remained the dominant LD transport mode (81%–96%), while Brownian-like and directed motion were consistently observed across all individuals (Fig. S7i). The above results demonstrate that PHYSIQ is not restricted to HeLa cells and can be broadly applied to diverse live-cell systems with distinct LD physiology and transport dynamics.

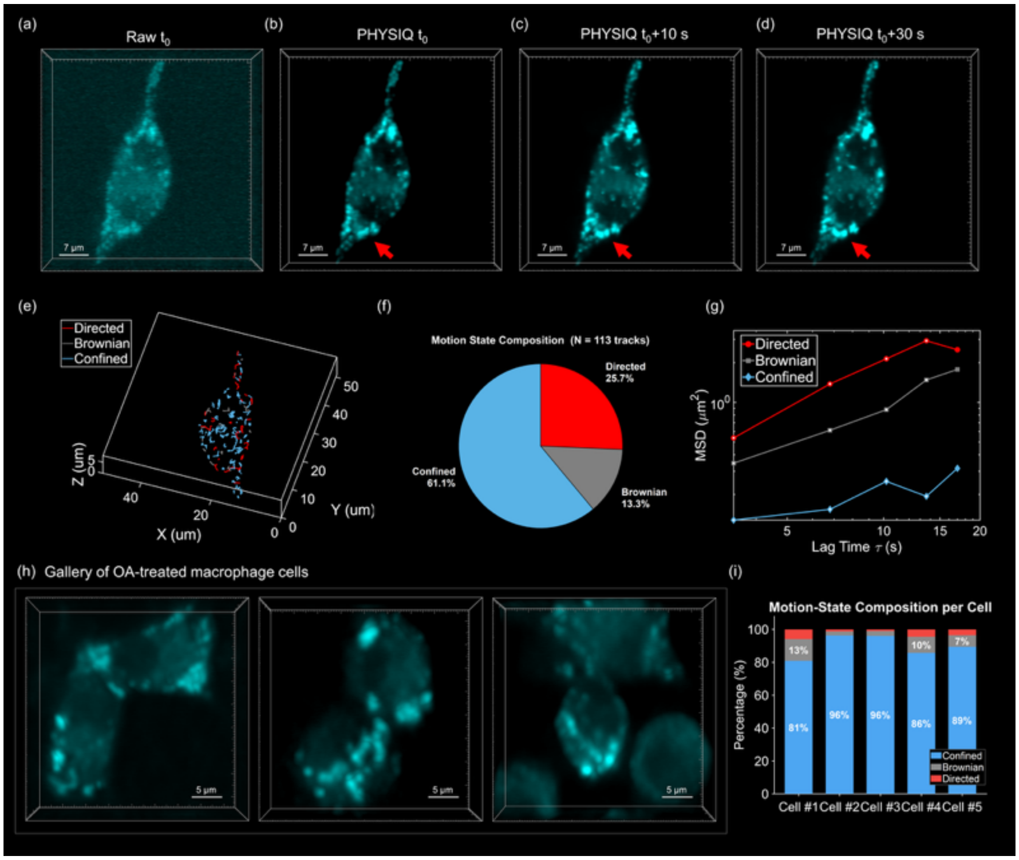

Figure S7. PHYSIQ enables volumetric tracking of lipid-droplet dynamics in live macrophages.

(a) Raw volumetric SRS image of a live macrophage at time t_0. (b–d) PHYSIQ-denoised volumetric images of the same cell at t_0, t_0 + 10 s, and t_0 + 30 s, showing clear visualization of intracellular LDs and their time-dependent redistribution. Red arrows highlight an example region with apparent long-range LD movement. Scale bars, 7 μm. (e) Representative 3D LD trajectories reconstructed from the PHYSIQ-denoised macrophage dataset and classified into directed (red), Brownian-like (gray), and confined (blue) motion states based on MSD scaling behavior. (f) Motion-state composition for the tracked trajectories in this representative macrophage cell (N = 113 tracks), showing that all three motility classes were observed, including a substantial directed-motion component. (g) MSD curves for representative directed, Brownian-like, and confined trajectory groups, illustrating distinct dynamic regimes in macrophage LD transport. (h) Gallery of additional OA-treated macrophage cells. Scale bars: 5 μm. (i) Population-level motion-state composition across 5 individual macrophage cells, showing the consistency of motility patterns across the cell population. Macrophage preparation. RAW264.7 murine macrophage-like cells were cultured in DMEM supplemented with high glucose, GlutaMAX, and 10% fetal bovine serum. Cells were seeded onto glass-bottom dishes and maintained at 37 °C in a humidified incubator with 5% CO_2 for 24 h before imaging. Immediately before SRS imaging, the culture medium was removed, and cells were washed twice with phosphate-buffered saline (PBS) at 37 °C and imaged in PBS medium. RAW264.7 cells were imaged under the same PHYSIQ-SRS conditions as HeLa cells.

Supplementary Note S10. Comparison between volumetric 3D tracking and conventional 2D analyses

To directly assess the necessity of volumetric imaging for accurate LD motion quantification, we compared kinetic parameters derived from full 3D tracking with those obtained using conventional 2D analyses. Representative 3D renderings of the same cell at two time points (t = 0 s and t = 161 s) are shown in Fig. S8a, b. Corresponding side-view projections reveal clear axial displacement of multiple LDs over time (red arrows), demonstrating that LDs are distributed across multiple focal planes and undergo substantial motion along the z-axis.

We first performed a direct comparison using the same set of tracked LD trajectories by calculating motion metrics either from the full 3D coordinates or from their 2D projected coordinates. This isolates the contribution of axial motion while preserving identical LD identities. As shown in Fig. S8c–e, inclusion of the z-coordinate increased the measured speed, maximum displacement, and MSD, consistent with the additional trajectory length contributed by out-of-plane motion. To further evaluate whether conventional single-plane analysis introduces focal-plane-dependent sampling bias, we independently performed 2D LD tracking at three representative axial positions (z = 3, 6, and 9 μm). These single-plane analyses yielded substantially different distributions of speed, maximum displacement, and MSD, with corresponding MSD curves varying markedly across imaging depth (Fig. S8c–f). Such layer-dependent variability is expected because each focal plane samples only a subset of LDs and fails to capture droplets moving into or out of the selected imaging plane.

Together, these comparisons demonstrate two fundamental limitations of conventional 2D approaches: projected trajectories systematically underestimate total displacement by excluding axial motion, while single-plane analyses introduce depth-dependent sampling bias by observing only a fraction of the intracellular LD 3D population. These results underscore the importance of volumetric PHYSIQ imaging for comprehensive and accurate quantification of LD 3D dynamics in living cells.

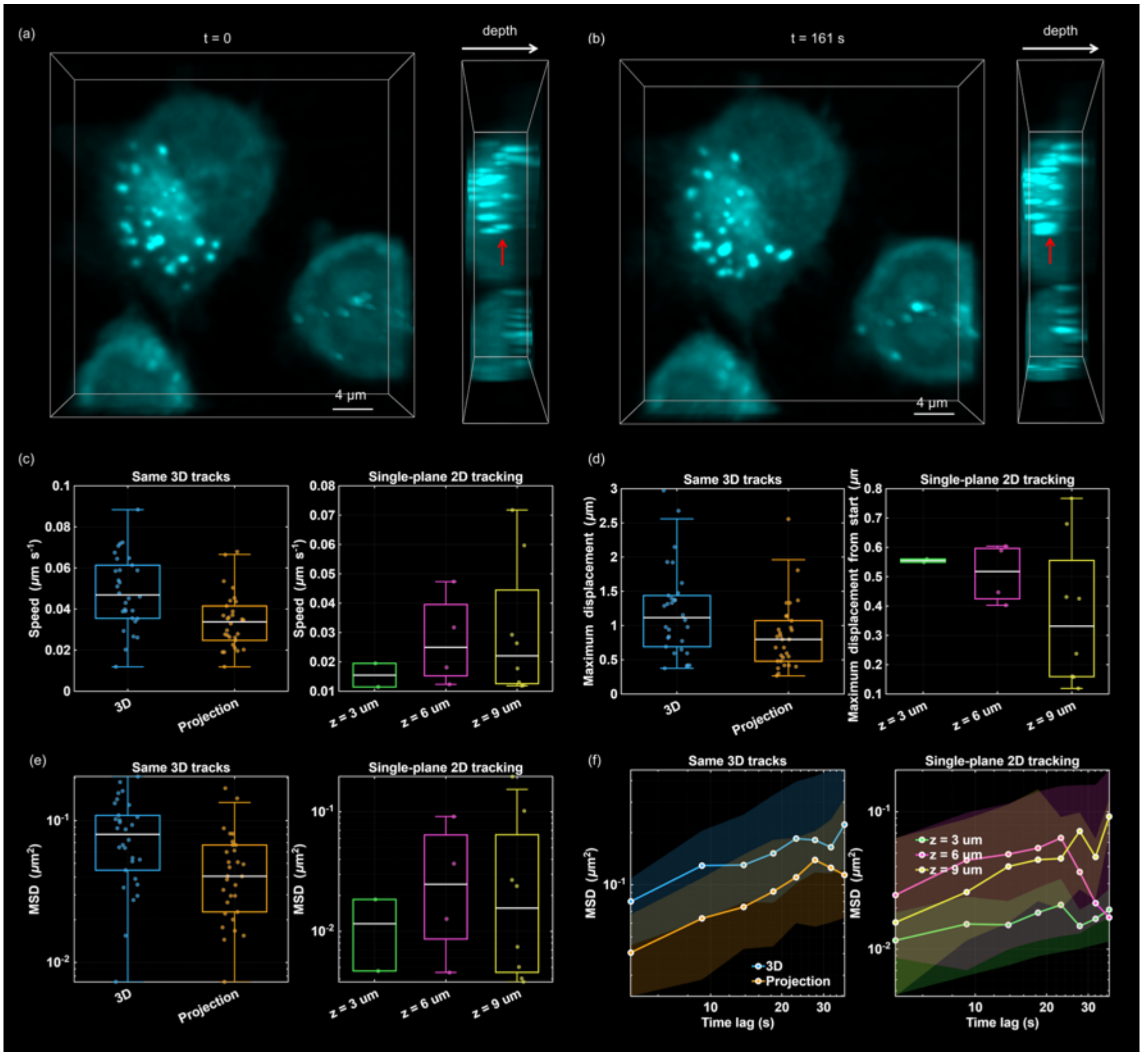

Figure S8. Volumetric tracking reveals axial LD motion and highlights discrepancies between 3D and 2D kinetic analyses.

(a, b) Representative 3D renderings of the same cell at t = 0 s (a) and t = 161 s (b). The side-view projections reveal clear axial displacement of LDs over time (red arrows), indicating that LDs are distributed across multiple depth planes and undergo z-directional motion. (c) Comparison of LD speed distributions. Left: kinetic parameters calculated from the same 3D tracks using full 3D coordinates or projected coordinates. Right: independent single-plane 2D tracking at representative axial layers, z = 3, 6 and 9 μm. (d-e) Comparison of MSD distributions (d) and maximum displacement (e) across the same analysis modes as in (c). (f) MSD curves derived from full 3D trajectories and their 2D projections, and from independent single-plane 2D tracking at z = 3, 6 and 9 μm. Shaded regions indicate the distribution range of MSD values across trajectories.

Supplementary Note S11. Optional dual-foci inference mode for accelerated volumetric imaging

Following training of the PHYSIQ denoising network using same-plane physics-paired in-phase/quadrature image pairs, the imaging system can be reconfigured into an optional dual-foci inference mode to accelerate volumetric acquisition. In this mode, the two excitation foci are intentionally separated along the axial direction to simultaneously acquire interleaved z-planes, effectively reducing the number of axial scan steps required to reconstruct a full three-dimensional volume. The pretrained PHYSIQ model is then directly applied to both channels for denoising, enabling faster volumetric imaging without additional model retraining.

We first validated this accelerated acquisition strategy using polymer beads suspended in water. As shown in Fig. S9a, the dual-foci configuration enabled rapid volumetric imaging, capturing clearly resolved bead displacements across consecutive volumes acquired only a few hundred milliseconds apart. This demonstrates that the approach supports video-rate 3D imaging of dynamic samples while preserving spatial fidelity.

Beyond single-band imaging, the dual-foci PHYSIQ framework can be extended to dual-color volumetric imaging by independently controlling the pump–Stokes temporal delay in each physics-paired channel. As demonstrated in Fig. S9b, this enables simultaneous acquisition of volumetric Raman images at the 2880 cm^{-1} and 2950 cm^{-1} vibrational bands with sub-second temporal resolution (840 *ms* per volume). These results establish that physics-paired acquisition is compatible with multiplexed chemical imaging using pretrained PHYSIQ model

PHYSIQ differs fundamentally from previously reported three-beam in-phase/quadrature architectures[6], in which the pump pulse is stretched substantially longer than the Stokes pulses. Such designs can reduce effective stimulated Raman interaction efficiency and complicate simultaneous alignment of both channels to the same vibrational resonance. By contrast, PHYSIQ is specifically engineered to preserve matched Raman contrast between paired channels at a single Raman peak, ensuring the signal correspondence required for Noise2Noise training while maintaining efficient signal generation and detection.

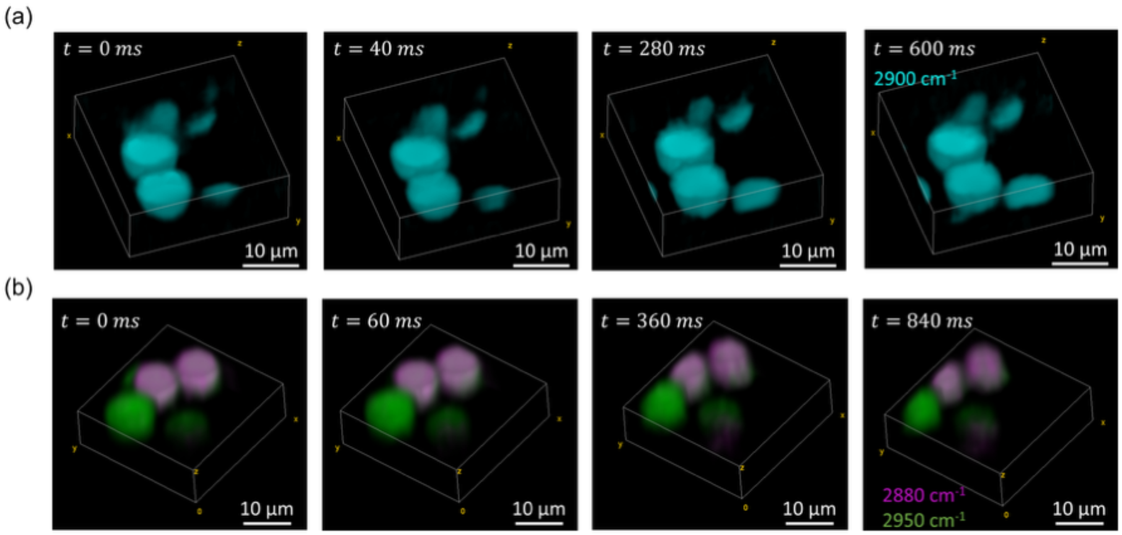

Figure S9. Time-lapse volumetric SRS imaging of polymer beads.

(a) Representative 3D renderings of 10 µm polystyrene (PS) beads imaged at 2900 cm^{-1} at t = 0, 40, 280, and 600 *ms*, showing clearly resolved displacements over consecutive volumes. (b) Dual-color 3D chemical renderings of a mixed microsphere sample containing 10 μm PS beads (magenta) and 10 µm poly(methyl methacrylate) (PMMA) beads (green), distinguished by spectral contrast at 2880 cm^{-1} and 2950 cm^{-1}. All volumes are displayed in the same viewing orientation. Scale bars, 10 μm. For the PMMA/PS measurements, pixel dwell time was 0.5 μs with a 256 × 256 pixel grid.

Supplementary Note S12. Power-scaling comparison of raw SRS and PHYSIQ imaging in HeLa cells

To directly assess whether the SNR achieved by PHYSIQ could be matched by simply increasing excitation power in conventional SRS, we performed a power-scaling study in live HeLa cells. Raw SRS volumes were acquired over a range of pump powers (5–70 mW), while maintaining identical pixel dwell time, image sampling, and detection settings. As shown in Fig. S10a, increasing the pump power progressively improved raw SRS contrast and reduced background noise. The Stokes power was held constant at 30 mW for all measurements, with a pixel dwell time of 4 μs. However, even at the maximum pump power tested (70 mW), the raw SRS images remained visibly noisier than the PHYSIQ reconstruction obtained under the standard low-power condition (Fig. S10b). Quantitative analysis showed that the raw SRS SNR increased only modestly, from 13.4 dB at 5 mW to 17.1 dB at 70 mW (Fig. S10c). Notably, the limited SNR gain observed between 40 and 70 mW suggests diminishing returns at higher excitation powers, likely due to detector saturation or reduced effective dynamic range under intense illumination. In contrast, PHYSIQ achieved an SNR of 26.2 dB under standard low-power imaging conditions, substantially outperforming even the highest-power raw SRS acquisition. These results demonstrate that PHYSIQ enhances effective image quality through physics-informed computational restoration, rather than through increased excitation power or photon dose.

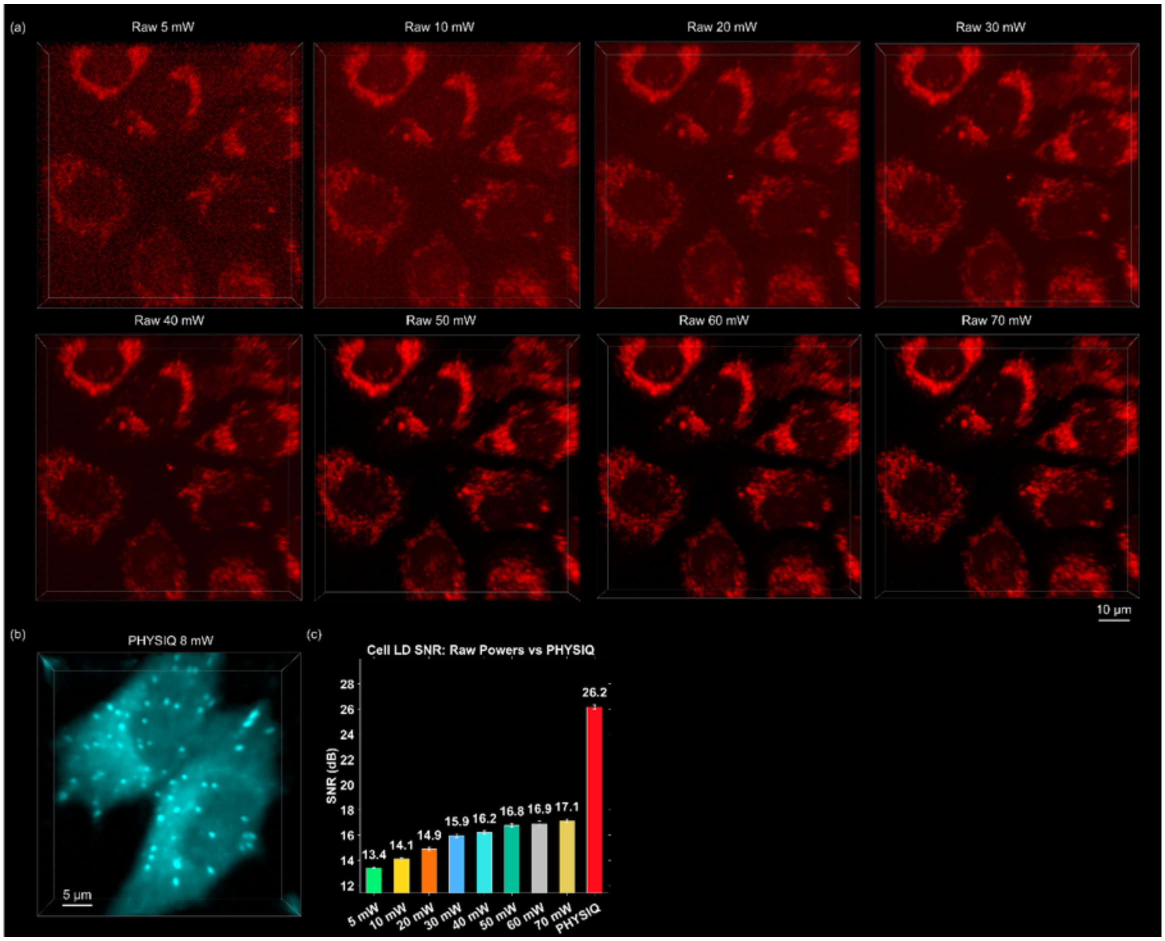

Figure S10. Power-scaling comparison of raw SRS and PHYSIQ imaging in HeLa cells.

(a) Representative raw SRS volumetric images of HeLa cells acquired at increasing pump powers from 5 to 70 mW. Although higher pump power improves image contrast in the raw SRS data, image quality remains inferior to that achieved by PHYSIQ reconstruction at low excitation power. **(b)** Representative PHYSIQ-reconstructed volumetric image of a HeLa cell acquired at 8 mW pump power, demonstrating enhanced image quality under substantially reduced excitation. **(c)** Quantitative SNR analysis across the pump-power series, highlighting the efficiency of PHYSIQ in recovering high-SNR images under low-power conditions. For all measurements, the Stokes power was fixed at 30 mW and the pixel dwell time was 4 μs; labels indicate pump power. Scale bars, 10 μm in **(a)** and 5 μm in **(b)**.

Supplementary Video 1.

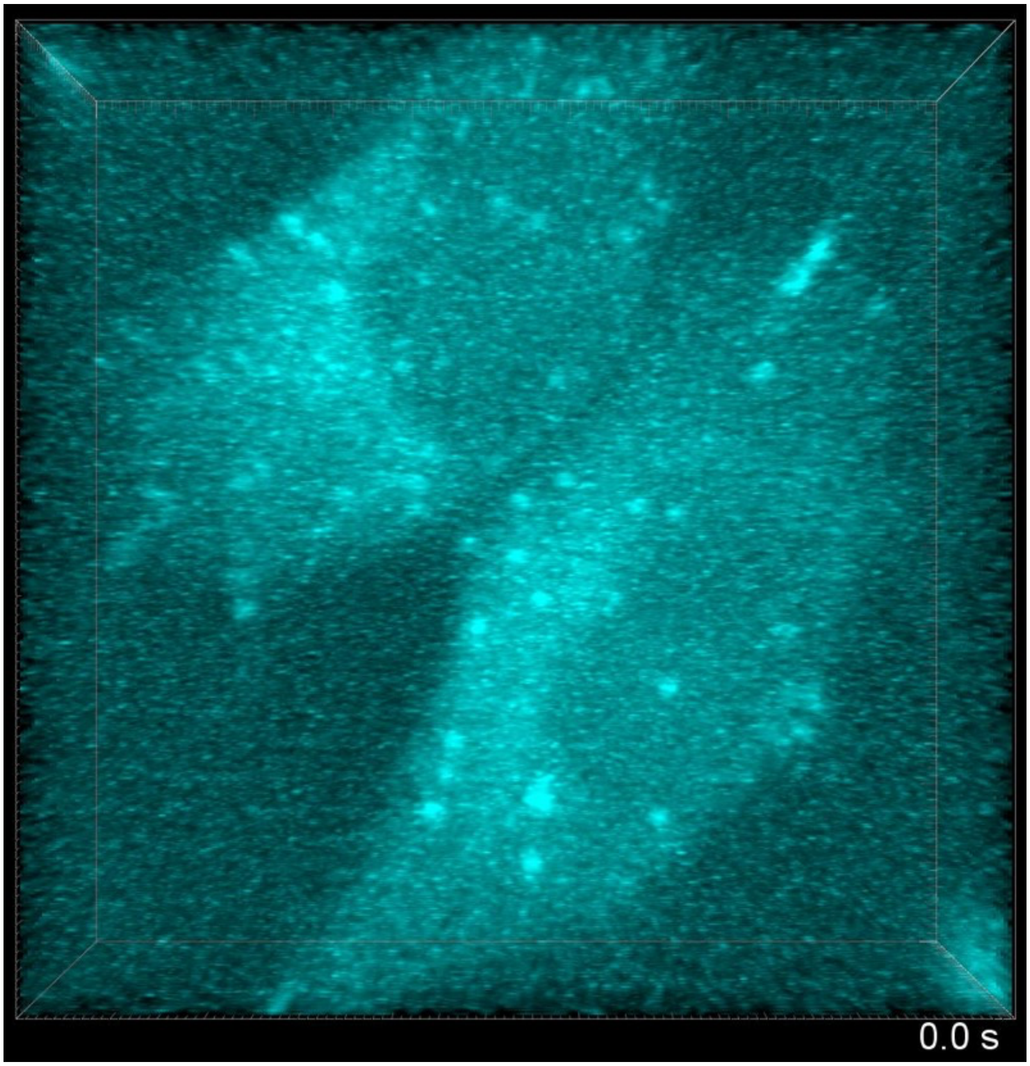

Video 1. Raw 3D SRS time-lapse imaging of a fixed HeLa cell acquired at 2850 cm^{-1}.

Despite the low SNR, LD positions remain largely unchanged over tens of seconds, supporting the use of the 10-volume mean as a surrogate reference for quantitative benchmarking.

Supplementary Video 2.

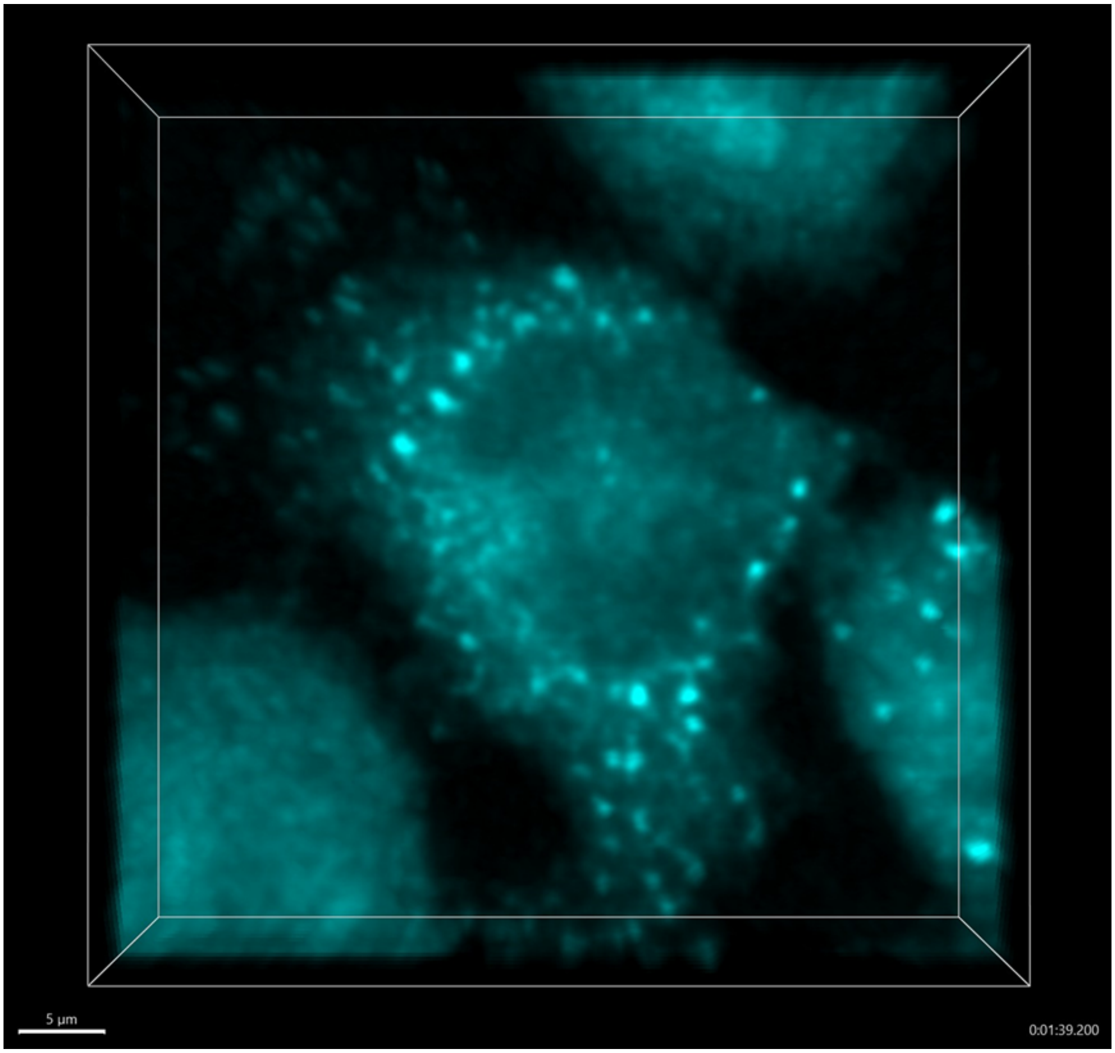

Video 2. PHYSIQ enables label-free 3D tracking of lipid droplets in live cells.

PHYSIQ-denoised volumetric SRS time-lapse imaging of a live HeLa cell under control conditions at 2850 cm^{-1} enables robust detection and three-dimensional trajectory reconstruction of LDs, facilitating label-free analysis of intracellular trafficking dynamics.

Supplementary Video 3.

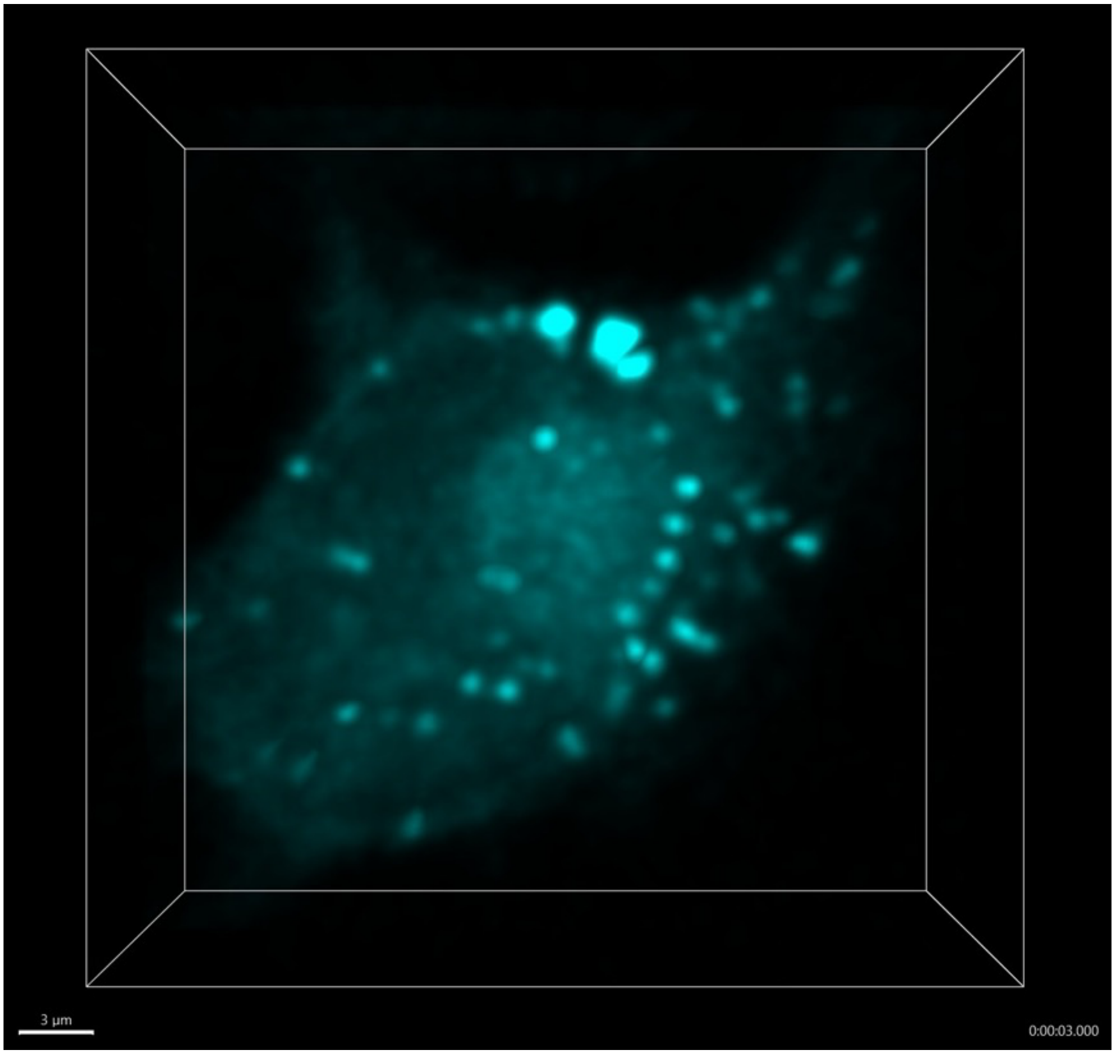

Video 3. Oleic acid loading enhances LD interactions and reprograms 3D trafficking dynamics

PHYSIQ-denoised volumetric SRS time-lapse imaging of an oleic acid (OA)-treated HeLa cell at 2850 cm^{-1} reveals increased LD contacts, fusion-like coalescence, and morphological remodeling, accompanied by altered intracellular trafficking dynamics in three dimensions.

Supplementary Video 4.

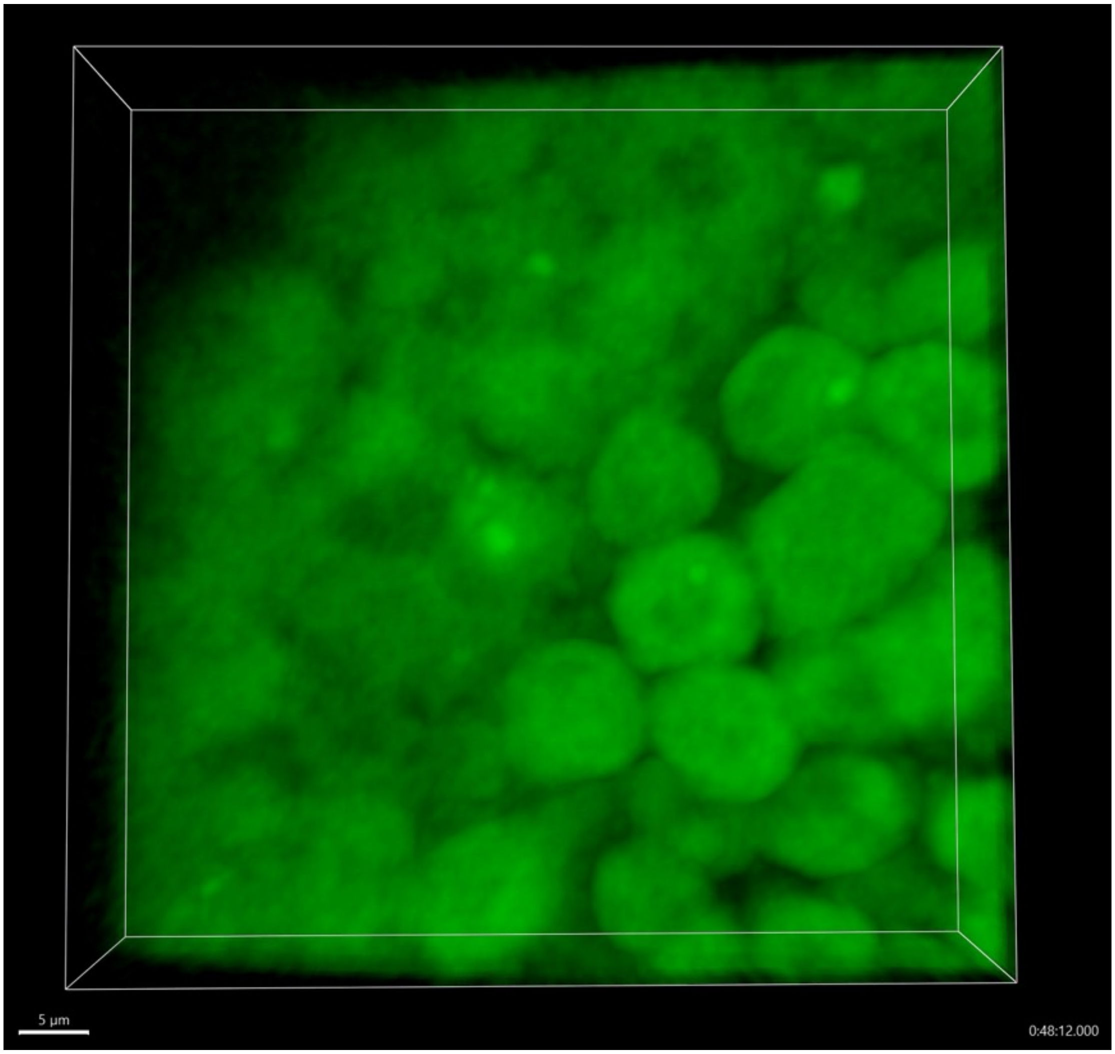

Video 4: Long-term in vivo volumetric SRS imaging of zebrafish embryo development enabled by PHYSIQ

Continuous PHYSIQ-based volumetric SRS imaging of a developing zebrafish embryo over 70 min, capturing morphogenetic dynamics and successive cell-division events in vivo. This dataset is presented as a qualitative proof-of-principle demonstration of long-term label-free volumetric imaging, rather than as a quantitative phototoxicity assessment.

References

1 Czarske, J. W. Statistical frequency measuring error of the quadrature demodulation technique for noisy single-tone pulse signals. *Measurement Science and Technology* **12**, 597-614 (2001).

2 Starshynov, I., Weimar, M., Rachbauer, L. M., Hackl, G., Faccio, D., Rotter, S. & Bouchet, D. Model-free estimation of the Cramér–Rao bound for deep learning microscopy in complex media. *Nature Photonics* **19**, 593-600 (2025).

3 Huang, J., Jin, Y., Yu, Z., Zhang, J., Jian, W. & Ren, Z. Equal distribution of lipid droplets in daughter cells is regulated by microtubules. *Cell Cycle* **22**, 1421-1433 (2023).

4 Chance, B., Cohen, P., Jobsis, F. & Schoener, B. Intracellular Oxidation-Reduction States in Vivo: The microfluorometry of pyridine nucleotide gives a continuous measurement of the oxidation state. *Science* **137**, 499-508 (1962).

5 Skala, M. C., Riching, K. M., Gendron-Fitzpatrick, A., Eickhoff, J., Eliceiri, K. W., White, J. G. & Ramanujam, N. In vivo multiphoton microscopy of NADH and FAD redox states, fluorescence lifetimes, and cellular morphology in precancerous epithelia. *Proceedings of the National Academy of Sciences* **104**, 19494-19499 (2007).

6 He, R., Xu, Y., Zhang, L., Ma, S., Wang, X., Ye, D. & Ji, M. Dual-phase stimulated Raman scattering microscopy for real-time two-color imaging. *Optica* **4**, 44-47 (2016).
